# Bis‐Dichlorosilyl Functionalized C_4_‐Cumulene With Unique Bonding Scenario

**DOI:** 10.1002/chem.202501556

**Published:** 2026-01-16

**Authors:** Saroj Kumar Kushvaha, Harsha S. Karnamkkott, Sangita Mondal, Paula A. M. Stark, Selvakumar Arumugam, Prakash Chandra Joshi, Sai Manoj N. V. T. Gorantla, Regine Herbst‐Irmer, Kartik Chandra Mondal, Dietmar Stalke, Herbert W. Roesky

**Affiliations:** ^1^ Institut Für Anorganische Chemie Georg‐August Universität Göttingen Germany; ^2^ Indian Institute of Technology Madras Chennai India; ^3^ Hylleraas Centre for Quantum Molecular Sciences Department of Chemistry University of Tromsø–The Arctic University of Norway Tromsø Norway

**Keywords:** cumulenes, EDA‐NOCV analysis, silicon chemistry, silylenes

## Abstract

This work reports the preparation and computational investigation of a bis‐dichlorosilyl functionalized C_4_‐cumulene (**2**), which was synthesized by employing Amidinato‐chlorosilylene, L(Cl)Si: (L = PhC(N*^t^* Bu)_2_). Compound **2** was characterized by single‐crystal X‐ray diffraction, mass spectrometry, and NMR spectroscopy. The stability, distribution of spin densities, and the nature of Si‐C bonds of **2** were studied by employing natural bond orbital (NBO) analysis, atoms in molecules (AIM), and energy decomposition analysis‐natural orbital for chemical valence (EDA‐NOCV). The EDA‐NOCV analysis showed that compound **2** possesses electron‐sharing covalent sigma and dative covalent sigma bonds (Si↔C and C→Si) between the Ph_2_C_4_ fragment in the anionic doublet state and silyl‐amidine groups in the cationic doublet state. This may be due to the electron‐deficient nature of olefin and electron‐rich N‐donating functional groups on silicon atoms. Compound **2** displays unprecedented chemical bonding in this class of compounds as predicted by EDA‐NOCV calculations. We have also compared the bonding situation of compound **2** with a previously reported compound **3**.

## Graphical abstract

Herein, we report a silylene‐functionalized C_4_‐cumulene (**2**) and investigate its bonding scenario employing computational methods such as NBO analysis, AIM, and energy decomposition analysis‐natural orbital for chemical valence (EDA‐NOCV). The EDA‐NOCV analysis showed that compound **2** prefers to possess electron‐sharing covalent sigma and dative covalent sigma bonds (Si↔C and C→Si) between Ph_2_C_4_ fragment in the anionic doublet state and silyl‐amidine groups in cationic doublet state. This may be due to the electron‐deficient nature of olefin and electron‐rich N‐donating functional groups on silicon atoms. Compound **2** displays unprecedented chemical bonding in this class of compounds as predicted by EDA‐NOCV calculations. We have also compared the bonding situation of compound **2** with a previously reported compound **3**.

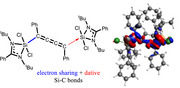

## 1 Introduction

**FIGURE 1:**
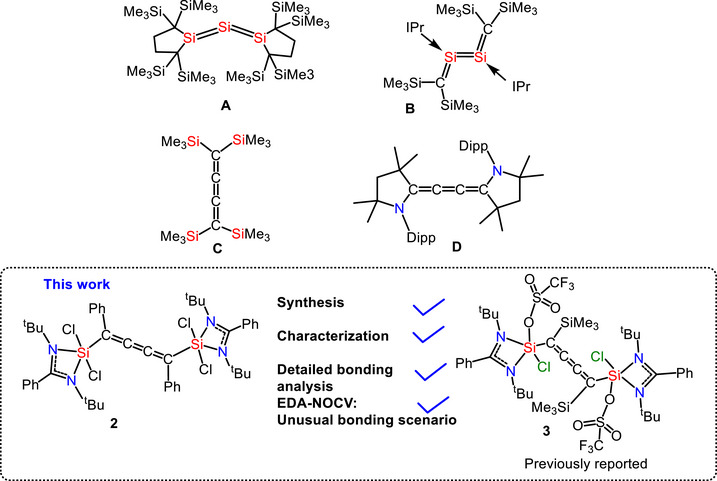
Selected examples of silicon‐centered spirocyclic compounds and the present work.

Silylenes are a highly reactive class of inorganic compounds typically featuring a divalent silicon atom with a lone pair of electrons and a vacant p‐orbital [1, 2, 3]. However, donor‐stabilized silylenes have been identified, where the electron pair from the nitrogen atom in the amidinate group is donated to the empty p‐orbital of the silicon atom of the silylene [4, 5, 6]. These silylenes lack the vacant p‐orbital, making them more stable than typical *N*‐heterocyclic silylenes (NHSi). Due to their similar electronic structures to carbenes, silylenes are considered their heavier congeners. Since the first report of NHSi, the chemistry of silylenes has advanced in various directions, with applications including small molecule activation [7, 8], the stabilization of low‐valent metal atoms [9], and their use as cross‐linkers in elastomers [10]. More recently, numerous reports have highlighted the use of stable silylenes in diverse areas of chemistry, including the isolation of multiply bonded silicon compounds [4, 5, 6, 11]. Since the isolation of the first silene (R_2_Si = CR_2_) and disilene (R_2_Si = SiR_2_), several other compounds with multiple‐bonded silicon compounds have been reported [12, 13, 14]. Kira et al. reported a stable silicon‐based allene analogue (**A**) with a formally *sp*‐hybridized silicon atom (Figure 1) [15]. The Si1‐Si2‐Si3 skeleton is not linear but is significantly bent with a bond angle of 136.49(6) Å and trans‐bent arrangement around Si1‐Si2 and Si3‐Si2 bonds, indicating that the bonding cannot be described by using simple *sp*‐hybridization. These unique structural features were first reported in the trisilaallene, **A**. Furthermore, a cumulene with C = Si = Si = C bonding arrangement (**B**) was reported by Cui and coworkers.[16] The formation of these allenes by heavier homologues of carbon is quite difficult, and it is not so commonly observed due to less effective pπ‐pπ overlapping as opposed to the carbon atom. However, multiple bonding with formally sp‐hybridized heavy group‐14 atoms can be achieved by taking advantage of the kinetic stabilization of bulky substituents. [17, 18] The stabilization of C_4_‐cumulene has also been achieved with the help of inorganic and organic substituents. Yasuyoka and coworkers reported C_4_‐cumulene (**C**), which is flanked by a comparatively smaller peripheral substituent, trimethylsilane.[19] The stabilization of C_4_‐cumulene (**D**) with an almost linear C = C = C = C skeleton has also been achieved using bulkier substituents of cyclic alkyl amino carbenes. [20, 21] Herein, we report the synthesis of C_4_‐cumulene (**2**) stabilized by amidinato‐chlorosilylene substituents. We have also performed detailed computational calculations to investigate bonding scenarios in compound **2** and our previously reported compound **3** [22]. The detailed computational investigation employing EDA‐NOCV revealed fascinating bonding situations, which were not reported earlier.

## 2 Results and Discussion

### 2.1 Synthesis

**SCHEME 1:**
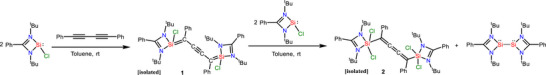
Proposed reaction scheme for the formation of (L)SiCl(Ph_2_C_4_)SiCl(L) (**1**) and (L)SiCl_2_(Ph_2_C_4_)SiCl_2_(L) (**2**) in the reaction mixture.

Amidinato‐chlorosilylene, L(Cl)Si: (L = PhC(N*^t^* Bu)_2_) and 1,4‐diphenylbuta‐1,3‐diyne were reacted at a 4:1 molar ratio in toluene at room temperature and stirred for 24 h to obtain a red‐colored reaction mixture (Scheme 1). The reaction mixture was filtered, and the resultant filtrate was dried under vacuum. The dried mass was extracted with diethyl ether. The ether solution was concentrated to about 8 mL and stored at room temperature for crystallization. Colorless single crystals of (L)SiCl_2_(Ph_2_C_4_)SiCl_2_(L) (**2**) were isolated after 15 days in 22% yield. When, we recorded the mass spectrometric data using reaction mixture, it was observed that it contains both compounds **1** and **2** (Figure S1). However, when reaction was performed in THF, compound **1** was obtained as a major product as dark red plates (Scheme S1) [22]. Compounds **1** and **2** were characterized by SC‐XRD, NMR, and mass spectrometry (Figures S1–S6 in Supporting Information). Single crystals of both **1** and **2** are highly sensitive to air loses color within a few minutes in solid state. They are stable under an inert atmosphere at rt for over a month. UV‐VIS measurements of **2** in THF showed a strong board band from 230 to 350 nm with a shoulder at 403 nm. Compound **1** is thermally stable till 210°C (Figure S13).

### 2.2 Structural Description

**FIGURE 2:**
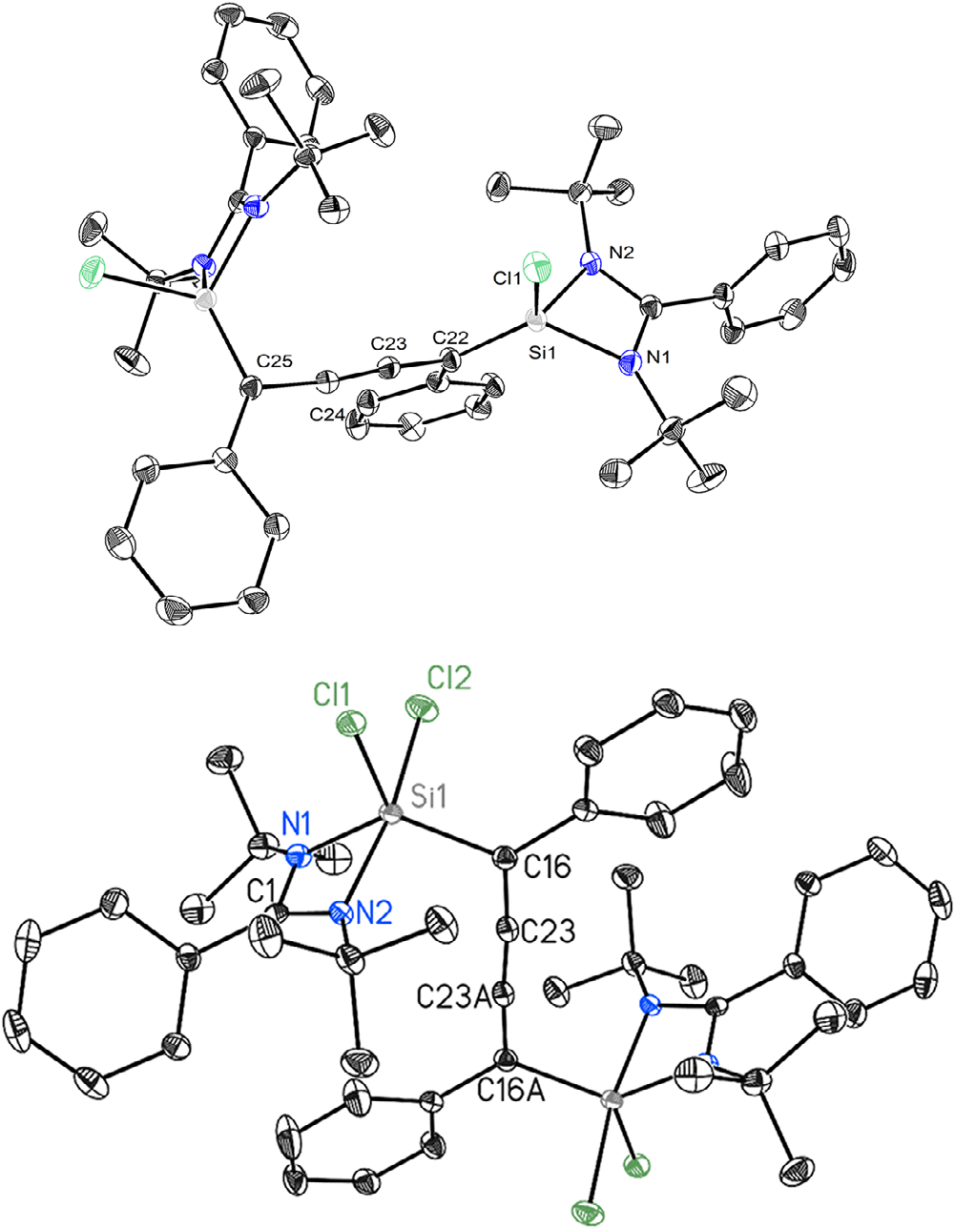
Molecular structures of compounds **1** (top) and **2** (bottom) with anisotropic displacement parameters at 50% probability level. Hydrogen atoms are omitted for clarity. Selected bond lengths [Å] and angles [°]: For **1**/**2**: Si1–C16 1.7582(13)‐ 1.7447(13)/1.8893(13), C16–C23 1.4227(17)‐1.4284(16)/1.3333(17), C23–C23A 1.2190(18)/1.265(2); for **2**: Si1–Cl1 2.1045(6), Si1–Cl2 2.1843(6), Si1–N1 1.8016(11), Si1–N2 1.9470(11), Si1–C16–C23 113.61(9), C16–C23–C23A 175.10(17), N1–Si1–N2 69.69(5).

The molecular structure of **1** was determined by single‐crystal XRD. Compound **1** crystallizes in the triclinic space group *P*‐1 and appears as orange‐red blocks (Figure 2). The Si1–N1 and Si1–N2 bond lengths are 1.8205(12) Å and 1.8097(11) Å, respectively, while the Si1–Cl1 bond measures 2.0329(7) Å. The Si1 atom also binds to a carbon atom (C22) with a bond length of 1.7582(13) Å. The structural motif features a linear arrangement at C23—C24 (1.2190(18) Å) typical of a carbon–carbon triple bond, with adjacent angle C24—C23—C22 measured at 167.87(13)°[22]. The coordination environment around the silicon center is pseudo‐tetrahedral, confirmed by the observed bond angles, and the crystal structure reveals a well‐defined molecular geometry supported by precise crystallographic data. The molecular structure of (L)SiCl_2_(Ph_2_C_4_)SiCl_2_(L) (**2**) was also confirmed by single‐crystal X‐ray diffraction studies (Figure 2). Compound **2** crystallizes in the monoclinic space group *P*2_1_/c with a half molecule [(L)SiCl_2_C(Ph)C] in the asymmetric unit. The Si1–C16 bond length, measured at 1.8893(13) Å, is similar to comparable bis‐amidinato‐chlorosilyl‐functionalized Ccumulene compounds [22] (Si–C: 1.864(2) Å and 1.830(4) Å), indicating a single bond.

The terminal bond lengths of the cumulenyl group C16–C23 in **2** are slightly longer at 1.3333(17) Å, while the C23‐C23A bond length in the same group is slightly shorter at 1.265(2) Å compared to the reported bond lengths in similar compounds [22] (C_terminal–_C 1.324(3) / 1.316(6) Å, C–C 1.276(4) / 1.278(9) Å). In addition, the angle between C16‐C23‐C23A, which is 175.10(17)°, deviates relatively more from linearity than the similar bis‐amidinato‐chlorosilyl‐functionalized C4 cumulene structures [22] (C–C–C: 179.1(3)° / 178.0(6)°) [22]. Selected bond parameters of **1** and **2** have been given in the caption of Figure 2 for the sake of comparison.

**FIGURE 3:**
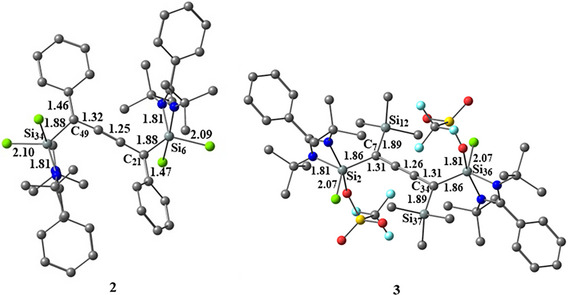
Optimized geometry of compounds **2** and **3** at PBE0‐D3(BJ)/def2‐TZVPP level of theory. Bond lengths in Angstroms and bond angles in degrees.

**FIGURE 4:**
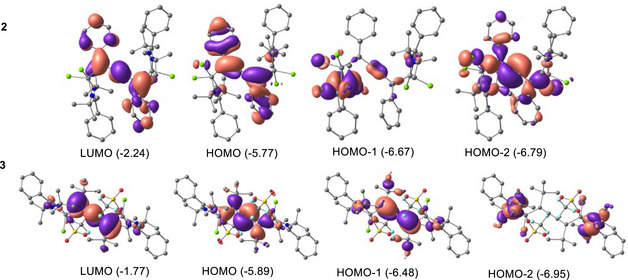
Molecular orbital pictures of the compounds **2** and **3** at PBE0‐D3(BJ)/def2‐TZVPP level of theory.

**FIGURE 5:**
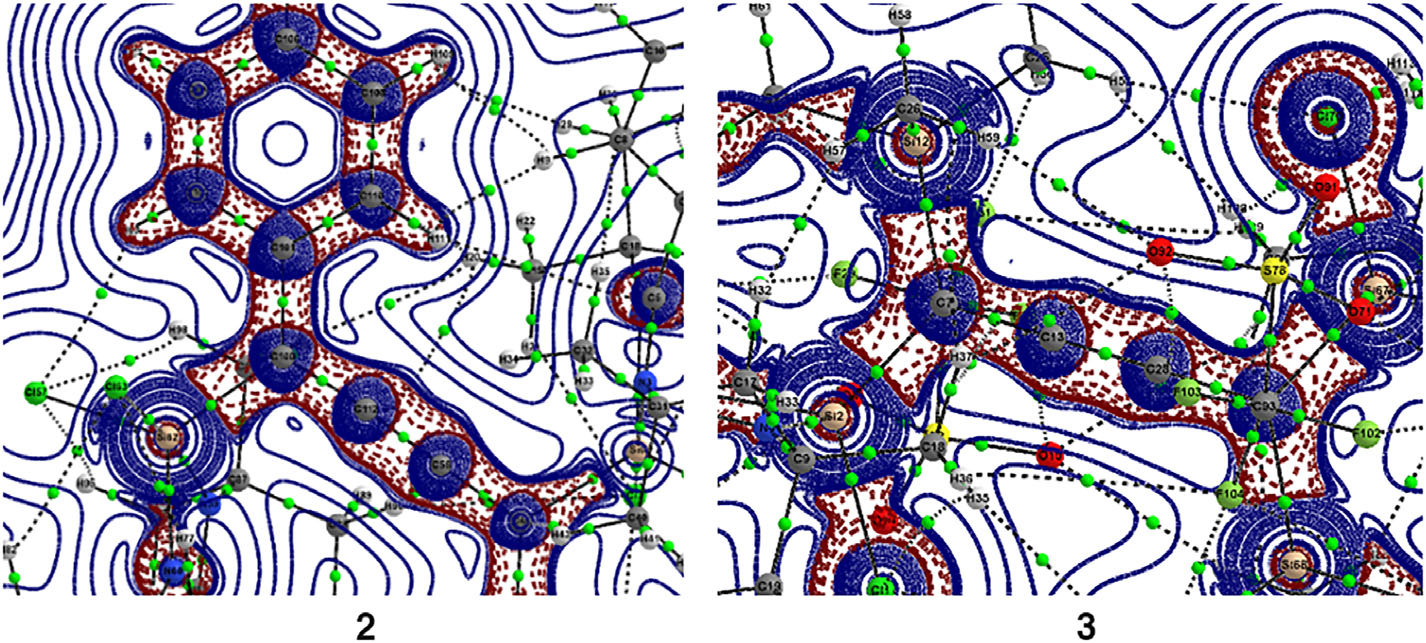
Contour plots of Laplacian distribution [∇^2^ *ρ*(r)] of compounds **2** and **3** in the Si‐C‐C plane. Solid blue lines indicate the areas of charge concentration (∇^2^ *ρ*(r) < 0), while dotted purple lines denote charge depletion (∇^2^ *ρ*(r) > 0). Solid lines connecting atomic nuclei (black) are the bond paths and small green spheres along the bond path are bond critical points (BCP). Those thick, solid blue lines separating the atomic basins indicate the zero‐flux surface crossing the molecular plane.

The DFT‐optimized geometry of compound **2** at PBE0‐D3(BJ)/def2‐TZVPP level of theory (Figure 3) shows good agreement between the computed and experimental bond lengths with very slight deviations (0.01‐0.02 Å). Additionally, we optimized and computationally investigated a synthetically reported compound **3**, with a similar cumulene backbone for better comparison and insights. We have performed natural bond orbital (NBO) and atoms in molecules (AIM) to understand the electronic properties and nature of the bonds of compounds **2** and **3**. The Wiberg bond indices of compounds **2** and **3** recommend a single bond character for Si−C bonds and a double bond character for C−C bonds, and the electron density of the Si−C bonds is highly polarized towards the C atoms (Table 1). The positive charge on the Si atoms and negative charge accumulation on the C atoms suggest charge migration from Si→C atoms. Nevertheless, the Cl atoms bonded to the Si atoms, too, take a pie of electron density, contributing to the high‐positive charge on the Si atoms. However, the net positive charge on Si atoms of compound **3** is slightly higher than that of **2**. The HOMO‐LUMO gap of 3.53 and 4.12 eV in compounds **2** and **3** indicates high electronic stability. The HOMO of compound **2** shows the delocalization of electron density between the phenyl rings through the carbon chain, while HOMO of **3** and HOMO‐2 of **2** reveal the Si−C σ‐type orbital interaction (Figure 4). The results from the AIM analysis of compounds **2** and **3** demonstrate a positive Laplacian (∇^2^ *ρ*(r)), negative total energy density, H(r) (Table 2), and a −G/V value of ∼0.6 [23, 24], inferring a partial covalent nature of the Si−C bond. A slightly polarized bond critical point (BCP) from the contour plot (Figure 5) also supports the same. The ellipticity (*ε*_BCP_ = *λ*_1_/*λ*_2–_1) values close to zero generally suggest either a single or triple bond character due to the cylindrical contours of electron density ρ [25, 26]. Nevertheless, with the Wiberg bond indices from the NBO analysis ruling out the possibility of a triple bond character, the current ellipticity values of compounds **2** and **3** can possibly imply a Si−C single bond character.

**TABLE 1:** NBO results of the Si−C bonds of compounds **2** and **3** at the PBE0‐D3(BJ)/def2‐TZVPP level of theory. Occupation number ON, polarization and hybridization of the bonds, and partial charges q.

Compound	Bond	ON	polarization and hybridization (%)		*WBI*	q Si	q C
**2**	Si_6_−C_21_	1.91	Si_3_: 26.5 s(35.8), p(63.2),d(1.0)	C_57_: 73.5 s(30.4), p(69.5), d(0.1)	0.66	Si_6_:1.734 Si_34_:1.715	C_21_: ‐0.502 C_49_: ‐0.476
	Si_34_−C_49_	1.91	Si_3_: 26.7 s(35.6), p(63.4),d(1.0)	C_49_: 73.3 s(30.3), p(69.6), d(0.1)	0.66		
**3**	Si_2_−C_7_	1.91	Si_2_: 26.2 s(36.2), p(63.0),d(0.8)	C_7_: 73.8 s(32.6), p(67.1), d(0.3)	0.70	Si_2_:1.971 Si_67_:1.971	C_7_: ‐0.953 C_63_: ‐0.953
	Si_67_−C_63_	1.91	Si_67_: 26.2 s(36.2), p(63.0),d(0.8)	C_63_: 73.8 s(32.6), p(67.1), d(0.3)	0.70		

**TABLE 2:** AIM results of compounds **2** and **3**.

Compound	Bond	*ρ*(r)	∇^2^ *ρ*(r)	*H*	*V*	*G*	ε
**2**	Si−C	0.121	0.169	−0.074	−0.185	0.111	0.066
	Si−C	0.118	0.151	−0.076	−0.194	0.118	0.061
**3**	Si−C	0.126	0.169	−0.081	−0.204	0.123	0.006
	Si−C	0.126	0.169	−0.081	−0.204	0.123	0.006

**SCHEME 2:**
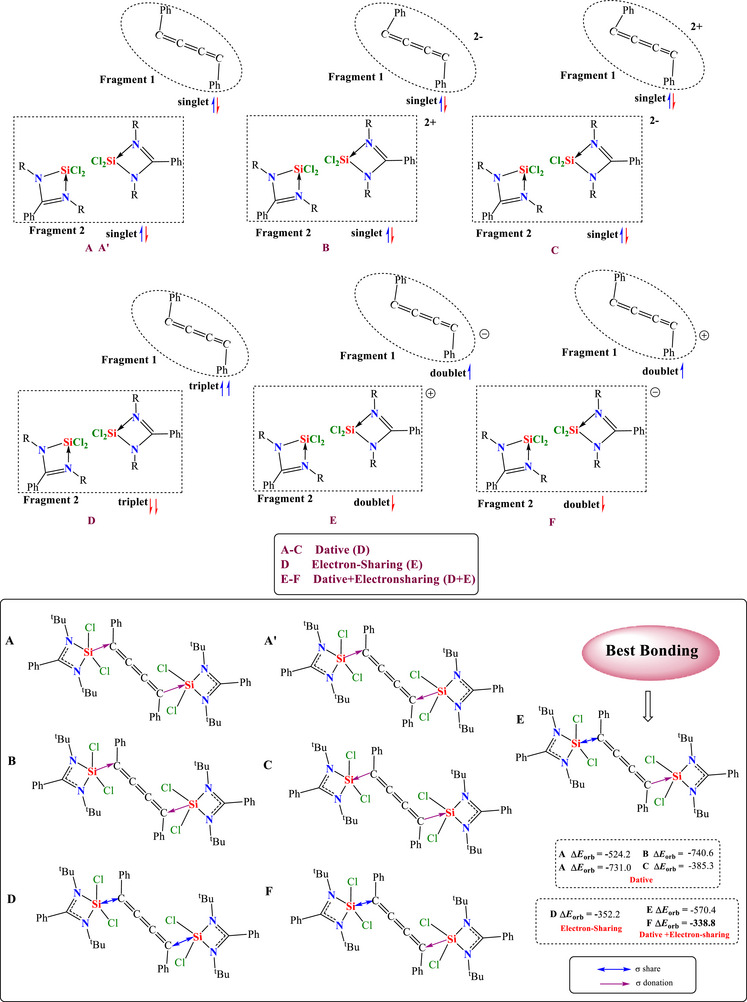
The fragmentation scheme with different charges and multiplicities (above) and corresponding bonding type representations (below) of compound **2**.

Though NBO and AIM analysis shed some light on the nature of Si−C bonding in compounds **2** and **3**, there still exists some ambiguity due to their inherent inability to distinguish the subtle differences in the electron‐sharing and dative covalent bonding. Hence, we carried out the energy decomposition analysis, coupled with natural orbitals for chemical valence (EDA‐NOCV), to get further insight into the nature of bonding. Scheme 2 shows different fragmentation schemes varying in terms of charges and multiplicities and corresponding bonding type representations of compound **2**. The bonding scenarios A and A' consider a dative Si→C, C→Si, and both Si→C type of bonding with L_2_(Cl_4_)Si_2_ and C_4_Ph_2_ fragments in the neutral singlet state. At the same time, B and C also represent a similar dative type of bonding but with doubly charged L_2_(Cl_4_)Si_2_ and C_4_Ph_2_ fragments in the singlet state. Whereas bonding type D demonstrates the Si↔C electron‐sharing bonding between the neutral L_2_(Cl_4_)Si_2_ and C_4_Ph_2_ fragments in a triplet state, and scenarios E and F show a mixture of Si↔C electron‐sharing and C→Si/Si→C dative bonding between the singly charged L_2_(Cl_4_)Si_2_ and C_4_Ph_2_ fragments in the doublet state (Scheme 2).

**FIGURE 6:**
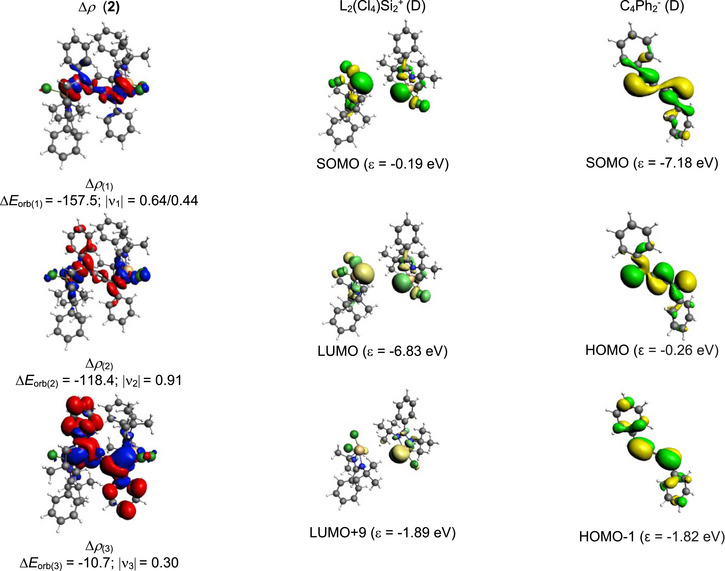
The shape of the deformation densities ∆*ρ*_(1)‐(3)_ that correspond to ∆*E*_orb(1)‐(3)_, and the associated fragments orbitals of compound **2** with charged L_2_Si_2_^+^and C_4_Ph_2_^−^ in doublet state at the PBE0‐D3(BJ)/TZ2P level. The isosurface value is 0.001 au for ∆*ρ*_(1, 2)_ and 0.0003 for ∆*ρ*_(3)_. The eigenvalues |ν_n_| give the size of the charge migration in e. The charge flow direction of the deformation densities is from red→blue.

**FIGURE 7:**
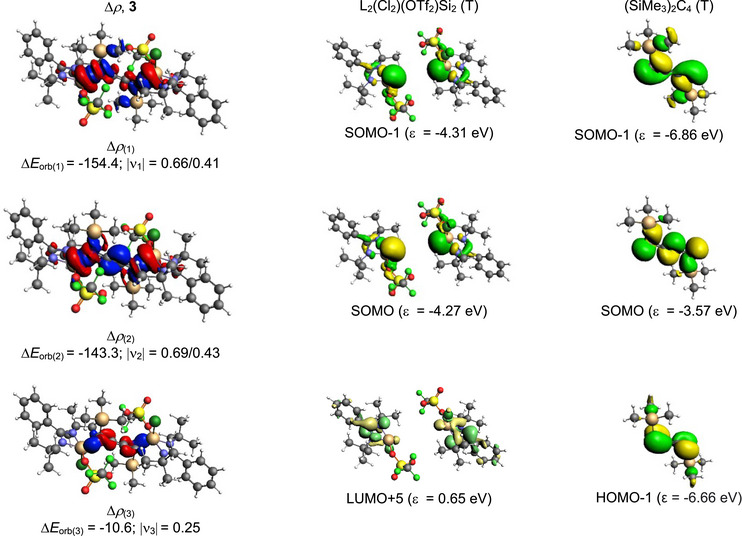
The charge flow direction of the deformation densities is from The shape of the deformation densities ∆*ρ*_(1)‐(2)_ that correspond to ∆*E*_orb(1)‐(2)_, and the associated fragments orbitals of compound **3** with neutral L_2_(Cl_2_)(OTf_2_)Si_2_ and (SiMe_3_)_2_C_4_ in triplet states at the PBE0‐D3(BJ)/TZ2P level. red→blue. The isosurface value is 0.001 au for ∆*ρ*_(1, 2)_ and 0.0003 for ∆*ρ*_(3)_. The eigenvalues |ν_n_| give the size of the charge migration in e. The charge flow direction of the deformation densities is from red→blue.

Among these possibilities, the bonding type E, representing a mixture of Si↔C electron‐sharing and C→Si dative bonding (Scheme 2), shows the lowest change in orbital interactions (∆*E*_orb_)[27, 28, 29, 30, 31, 32] and, therefore, is conferred as the best bonding scenario in compound **2** (Table 3). Further analysis suggests that the electrostatic contribution (53.1%) dominates the total attractive forces responsible for the stability of the bond, while orbital (covalent) and dispersion contributions also play a significant part (Table 4). Pairwise breakdown (Table 4) suggests a major share of orbital interactions (∆*E*_orb_) comes from Si↔C electron‐sharing σ interaction (46.5%) followed by C→Si σ electron donation (35%). Interestingly, a minor 3.5% contribution comes from the C→Si π electron donation in compound **2**. Figure 6 illustrates the deformation density pictures corresponding to the pairwise orbital interactions of compound **2**. On the contrary, the bonding analysis of compound **3**, carried out on similar lines to compound **2**, indicates that the nature of Si−C bonding can be explained both in terms of Si↔C electron‐sharing (bonding type D, Scheme 2) and a mixture of Si↔C electron‐sharing and C→Si dative bonding (bonding type E, Scheme 2). This is due to the slight difference (4.2 kcal/mol) in the change in orbital interactions (∆*E*_orb_) between the bonding possibilities D and E in compound **3** (Table 3). The breakdown of the total orbital contributions of compound **3** into pairwise interactions (Table 4) demonstrates two major Si↔C electron sharing contributions, as shown in Figure 7.

**TABLE 3:** The EDA‐NOCV results of L_2_(Cl_4_)Si_2_−C_4_Ph_2_ and L_2_(Cl_2_)(OTf_2_)Si_2_−C_4_(SiMe_3_)_2_ bonds of compounds **2** and **3** respectively at the PBE0‐D3(BJ)/TZ2P level, where D is dative, E is e‐sharing, and D+E represents the combination of dative + sharing bond formation. Energies are in kcal/mol. The most favorable bonding scheme is given by the smallest ∆*E*_orb_ value and is represented in bold. (Bonding schemes A‐F in square brackets).

Compound	Bond type	Fragments	∆*E*_int_	∆*E*_pauli_	∆*E*_dis_	∆*E*_elec_	∆*E*_orb_
**2**	**D**	L_2_(Cl_4_)Si_2_ (S) + C_4_Ph_2_ (S) [A]	−215.5	645.3	−27.3	−309.4	−524.2
		L_2_(Cl_4_)Si_2_ (S) + C_4_Ph_2_ (S) [A']	−685.1	667.5	−27.3	−594.3	−731.0
		L_2_(Cl_4_)Si_2_^2+^ (S) + C_4_Ph_2_^2−^ (S) [B]	−557.2	508.7	−27.3	−653.5	−385.3
		L_2_(Cl_4_)Si_2_^2−^ (S) + C_4_Ph_2_^2+^(S) [C]	−537.0	576.1	−27.3	−345.2	−740.6
	**E**	L_2_(Cl_4_)Si_2_ (T) + C_4_Ph_2_ (T) [D]	−215.8	482.9	−27.3	−319.2	−352.2
	**D+E**	L_2_(Cl_4_)Si_2_^+^ (D) + C_4_Ph_2_^−^ (D) [E]	−334.2	445.8	−27.3	−414.1	**−338.8**
		L_2_(Cl_4_)Si_2_^−^ (D)+ C_4_Ph_2_^+^ (D) [F]	−353.9	639.4	−27.3	−395.7	−570.4
**3**	**D**	L_2_(Cl_2_)(OTf_2_)Si_2_ (S) + C_4_(SiMe_3_)_2_ (S) [A]	−205.2	651.3	−30.4	−304.4	−521.6
		L_2_(Cl_2_)(OTf_2_)Si_2_^2+^ (S) + C_4_(SiMe_3_)_2_^2−^ (S) [B]	−609.8	527.6	−30.4	−680.6	−425.8
		L_2_(Cl_2_)(OTf_2_)Si_2_^2−^ (S) + C_4_(SiMe_3_)_2_^2+^(S) [C]	−677.0	676.7	−30.4	−600.2	−723.2
	**E**	L_2_(Cl_2_)(OTf_2_)Si_2_ (T) + C_4_(SiMe_3_)_2_ (T) [D]	−220.8	479.0	−30.4	−316.6	**−352.6**
	**D+E**	L_2_(Cl_2_)(OTf_2_)Si_2_^+^ (D) + C_4_(SiMe_3_)_2_^−^ (D) [E]	−370.2	446.4	−30.4	−429.4	**−356.8**
		L_2_(Cl_2_)(OTf_2_)Si_2_^−^ (D) + C_4_(SiMe_3_)_2_^+^ (D) [F]	−1056.2	599.8	−30.4	−417.4	−1208.2

**TABLE 4:** The pairwise interactions from EDA‐NOCV results of L_2_(Cl_4_)Si_2_−C_4_Ph_2_ and L_2_(Cl_2_)(OTf_2_)Si_2_−C_4_(SiMe_3_)_2_ bonds of compounds **2** and **3** at the PBE0‐D3(BJ)/TZ2P level. Energies are in kcal/mol.^[a]^ Values in the parentheses show the contribution to the total attractive interactions ∆*E*_elec_ + ∆*E*_orb_ +∆*E*_disp_^[a]^, and the ^[b]^ values in the parentheses show the contribution to the total orbital interactions.

Energy	Interaction	L_2_(Cl_4_)Si_2_^+^ (D) + C_4_Ph_2_^−^ (D) 2	Interaction	L_2_(Cl_2_)(OTf_2_)Si_2_ (T) + C_4_(SiMe_3_)_2_ (T) 3
∆*E*_int_		−334.2		−220.8
∆*E*_Pauli_		445.8		479.0
∆*E*_disp_^[a]^		−27.3 (3.5%)		−30.4 (4.3%)
∆*E*_elstat_^[a]^		−414.1 (53.1%)		−316.6(45.3%)
∆*E*_orb_^[a]^		−338.8 (43.4%)		−352.6 (50.4%)
∆*E*_orb(1)_^[b]^	L_2_(Cl_4_)Si_2_^+^ ↔ C_4_Ph_2_^−^ σ e^−^ share	−157.5 (46.5%)	L_2_(Cl_2_)(OTf_2_)Si_2_↔ C_4_(SiMe_3_)_2_ σ e^−^ share	−154.4 (43.8%)
∆*E*_orb(2)_^[b]^	L_2_(Cl_4_)Si_2_^+^← C_4_Ph_2_^−^ σ e^−^ donation	−118.4 (35.0%)	L_2_(Cl_2_)(OTf_2_)Si_2_↔ C_4_(SiMe_3_)_2_ σ e^−^ share	−143.3 (40.6%)
∆*E*_orb(3)_^[b]^	L_2_(Cl_4_)Si_2_^+^← C_4_Ph_2_^−^ π e^−^ donation	−10.7 (3.1%)	L_2_(Cl_2_)(OTf_2_)Si_2_← C_4_(SiMe_3_)_2_ e^−^ donation	−10.6 (3.0%)
∆*E*_orb(rest)_^[b]^		−52.2 (15.4%)		−44.3 (12.6%)

**FIGURE 8:**
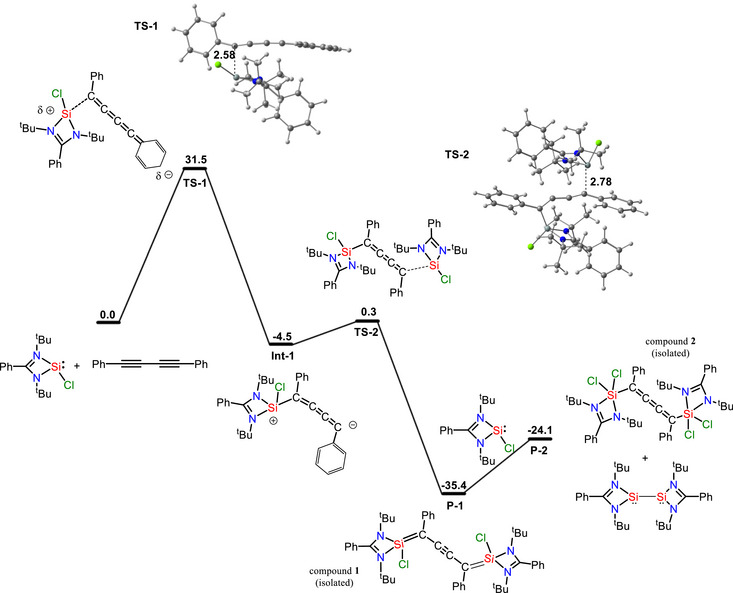
The free energy profile of reaction mechanism towards the formation of products **1** and **2** at PBE0‐D3BJ/6‐31+G** level in gas phase.

We have computationally investigated the reaction mechanism for the formation of products **1** and **2** using DFT methods (PBE0‐D3BJ/6‐31+G**, CPCM‐Toluene) (Figure 8). The first step involves the attack of one L(Cl)Si unit on the C_4_Ph_2_ unit resulting in the formation of a mono Si coordinated intermediate **Int‐1** via a transition state **TS1** with a barrier of 31.5 kcal/mol (Figure 8). The high energy barrier of this step makes it rate limiting. In the next stage product **P‐1** (compound **1**) is produced by the subsequent interaction of the second L(Cl)Si unit with **Int‐1** via transition state **TS2** with a relatively low energy barrier of 0.3 kcal/mol (Figure 8). Product **P‐1** again reacts with 2 moles of L(Cl)Si leading to the formation of product **P‐2** and LSi dimer. Despite many attempts, we failed to locate the transition state for the transfer of chlorines from L(Cl)Si to **P‐1** forming the product **P‐2** (compound **2**). The instant formation of an LSi dimer and quick transfer of Cl atoms to **P‐1** might have rendered the location of the transition state obscure.

**FIGURE 9:**
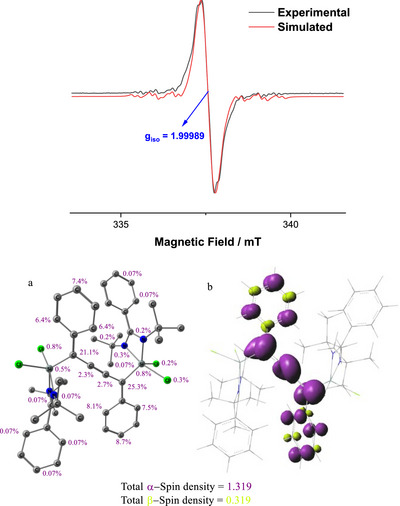
(Top) X‐band EPR spectrum (black) of 2^•−^ at room temperature in THF solvent. Red and black lines represent the simulated and experimental spectra of SM133 complex and the simulation is done using the EasySpin program. [giso = 1.99989, LWPP (Gaussian broadening) = 0.0590046 mT, LWPP (Lorentzian broadening) = 0.0109743 mT, A(13C type‐1) = 4.5 MHz, A(13C type‐2) = 71.05 MHz, A(13C type‐3) = 14.7 MHz A(13C type‐4) = 14.7 MHz, A(13C type‐5) = 18.62 MHz, X‐band experimental frequency = 9.449272 GHz]. (Bottom) The α‐spin density percentage (a) and the spin density plot (b) of 2^•−^ (q = ‐1 and S = ½) in doublet state at PBE0‐D3(BJ)/def2‐TZVPP level of theory. The isosurface value of the spin density plot is 0.003 au, purple color denotes the α‐spin density and green color denotes the β‐spin density.

The cyclic voltammetry measurements (Figure S11) in THF showed an irreversible process, suggesting the formation of 2^•−^. The X‐band EPR spectrum of 2^•−^ was recorded in THF solvent at room temperature [2 + KC_8_ → 2^•−^] (Figure 9). The spectrum is isotropic in nature with a g‐value of 1.99989. Based on both the spin density distribution and molecular symmetry, only five out of ten carbon nuclei were considered for the simulation. The hyperfine splitting arises from the interaction between the unpaired electron on the carbon atom and the ^13^C nucleus (*I* = ½). As shown in the spin density plot, the C1‐type carbon atom on the aliphatic chain, having a spin density of 2.3–2.7%, exhibits a coupling constant of 4.5 MHz. The C2 carbon, which is attached to both the aliphatic chain and the aromatic ring, has a higher spin density of 21.1–25.3% and exhibits a hyperfine splitting of 71.05 MHz. This clearly indicates that a higher spin density leads to a stronger electron–nucleus spin coupling interaction, resulting in a larger hyperfine coupling constant. On the phenyl ring, the ortho carbons (C3 and C4) with spin densities of 6.4–8.1% and 6.4–7.5%, respectively, exhibit splitting values of 14.7 MHz and 14.7 MHz. The para carbon (C5), with a spin density of 7.4–8.7%, shows a coupling constant of 18.62 MHz in the spectrum. This EPR measurement, simulation, and computation of Mulliken spin densities (Figure 9) further support the findings of EDA‐NOCV results rationalizing the electron‐deficient nature of PhC_4_Ph unit in 2. Addition of an external electron to 2 leads to formation of 2^•−^ having maximum spin densities are distributed along C4 chain and also on Ph rings.

## 3 Conclusion

In conclusion, compound (L)_2_SiCl_2_‐(Ph_2_C_4_)(SiCl_2_(L) (**2**), which can be regarded as 1,4‐disilyltetrachloro‐bis‐amidinate having a Si‐C = C = C = C‐Si backbone [22], was synthesized by reacting amidinato chlorosilylene, L(Cl)Si: (L = PhC(N*^t^* Bu)_2_). with 1,4‐diphenylbuta‐1,3‐diyne in toluene. The nature of the chemical bond between Ph‐CCCC‐Ph and two (L)SiCl_2_ units was investigated in detail with NBO, QTAIM, and EDA‐NOCV analysis and compared with our previously reported compound **3**. The EDA‐NOCV method suggested that the bonding in compound **2** can be best described in terms of a mixture of electron‐sharing and dative covalent bonds between the negatively charged central Ph‐CCCC‐Ph fragment and the positively charged {(L)_2_SiCl_2_}_2_ fragments, whereas bonding in compound **3** prefers to be described both as Si↔C electron‐sharing and a mixture of Si↔C electron‐sharing and C→Si dative bonding. The chemical bonding of silyl functionalized C_4_‐cumulene (**2**) is unprecedented.

## 4 Experimental Section

### 4.1 Crystal Data

The datasets were collected using a Bruker D8 three‐circle diffractometer, equipped with a Bruker Photon III C7 CMOS detector and an INCOATEC microfocus source (Mo K_α_ radiation) with INCOATEC Quazar mirror optics. The data integration process was executed using SAINT [33] and a multiscan absorption correction was applied via SADABS [34]. The structures were solved by SHELXT [35] and refined on *F* ^2^ using SHELXL [35] in the graphical user interface ShelXle [36]. All hydrogen atoms were placed according to geometrical criteria and refined with a riding model.

Crystal data for **1** at 100(2) K: C_46_H_56_Cl_2_N_4_Si_2_, *M*_r_ = 792.02 g mol^−1^, 0.26 × 0.13 × 0.10 mm, Triclinic, *P‐1*, *a =* 10.695(2) Å, *b =* 12.850(2) Å, *c =* 17.05(3) Å, *α* = 69.50(2), *β =* 81.06(2), *γ* = 81.83(2), *V =* 2158.4(9) Å^3^, *Z =* 2, *μ*(MoK_α_) *=* 0.243 mm^−1^, *θ*_max_ = 28.705°, 91603 reflections measured, 11096 independent (*R*_int_ *=* 0.0381), *R*_1_ *=* 0.0381 [*I > 2σ*(*I*)], *wR*_2_ *=* 0.1044 (all data), *Δρ*_max_ */Δρ*_min_ = 0.503 and ‐0.257 *e* Å^−3^, CCDC: 2503170.

Crystal data for **2** at 100(2) K: C_46_H_56_Cl_4_N_4_Si_2_, *M*_r_ = 862.92 g mol^−1^, 0.253×0.083×0.074 mm, Monoclinic, *P*2_1_ */c*, *a =* 10.373(2) Å, *b =* 18.725(4) Å, *c =* 12.465(3) Å, *β =* 109.86(2), *V =* 2277.1(9) Å^3^, *Z =* 2, *μ*(MoK_α_) *=* 0.349 mm^−1^, *θ*_max_ = 26.388°, 176882 reflections measured, 4660 independent (*R*_int_ *=* 0.0285), *R*_1_ *=* 0.0267 [*I > 2σ*(*I*)], *wR*_2_ *=* 0.0663 (all data), *Δρ*_max_ */Δρ*_min_ = 0.380 and ‐0.221 *e* Å^−3^, CCDC: 2400672.

## Author Contributions

S. K. K. conceptualized and designed the project, synthesized compounds 1‐2, collected XRD data, Performed mass spectrometric mesuremnets, and prepared original draft, P. C. J., S. M. and S. A. synthesized starting material, collected NMR, UV, EPR, CV and IR data, SMNVT Gorantla and H.S. performed DFT calculations, P. M. A. S and R. Herbst‐Irmer solved crystal structures. H. W. R., D. S. and K. C. M. secured funding, conceived the ideas and also wrote the manuscript.

## Conflicts of Interest

There are no conflicts to declare.

## Supporting information



Please see supporting information (SI) for NMR, crystallographic data and computational details. **Supporting File1**: chem70652‐sup‐0001‐SuppMat.docx

**Supporting File2**: chem70652‐sup‐0002‐SuppMat.Zip
