# Supplementary material for: Bis‐Dichlorosilyl Functionalized C4‐Cumulene With Unique Bonding Scenario
Source: Chemistry. 2026 Jan 16;32(12):e01556. doi: 10.1002/chem.202501556 (PMC13037364; doi:10.1002/chem.202501556)
Supplement: Supplementary file 1 — Please see supporting information (SI) for NMR, crystallographic data and computational details. Supporting File1: chem70652‐sup‐0001‐SuppMat.docx [file CHEM-32-e01556-s002.pdf]

Supporting Information

Bis-dichlorosilyl functionalized C_4-cumulene with unique bonding scenario

Saroj Kumar Kushvaha[a], Harsha S. Karnamkkott[b], Sangita Mondal[b], Paula Kallenbach[a], Sai Manoj N. V. T. Gorantla*[c], Selvakumar Arumugam, Prakash Chandra Joshi, Regine Herbst-Irmer[a], Kartik Chandra Mondal*[b], Dietmar Stalke*[a], Herbert W. Roesky*[a]

1. Experimental section

1.1. Materials and methods

All the chemicals were purchased from commercially available sources and were used as received. The solvents were dried by stirring over Na-K alloy for several days. All manipulations were carried out using standard Schlenk and glovebox techniques under high purity dinitrogen gas atmosphere. Deuterated NMR solvents C_6D_6/ THF-D_8 were dried by stirring it for 2 days over Na/K alloy followed by distillation in vacuum and degassed. ^{1}H, ^{13}C and ^{29}Si NMR spectra were recorded on Bruker Avance 400 or 500 MHz, 100 MHz and 99 MHz NMR spectrometers respectively and referenced to the resonances of the solvent used. Amidinato-silylene chloride, L(Cl)Si: (L = PhC(N^{t}Bu)$_2$) [1-2] was synthesized as per the procedure reported in the literature.

1.2. Synthetic procedure of 1:

In a 50 mL Schlenk flask, 65.5 mg (0.22 mmol) of amidinato-chlorosilylene was dissolved in 10 mL of tetrahydrofuran (THF) under an inert argon atmosphere, yielding a clear yellowish solution. To this solution, 1,4-diphenylbuta-1,3-diyne (14 mg, 0.06 mmol) was added, maintaining an approximate 4:1 molar ratio of silylene to diyne. The reaction mixture turned dark brown within one minute and was allowed to stir at room temperature for 16 h. The resulting clear brown solution was concentrated under reduced pressure to approximately 2 mL, followed by slow addition of 2 mL of toluene. The solution was left undisturbed for crystallization. A precipitate-like appearance was observed immediately, and after three days, red block-shaped crystals of **1** suitable for single-crystal X-ray diffraction (SC-XRD) analysis were obtained. **Yield:** 35 % (with respect to 1,4-diphenylbuta-1,3-diyne) The compound **1** was also characterized by ^{1}H and ^{13}C NMR; however, we did not observe the 29Si signals due to low- solubility of the compound and low-sensitivity of ^{29}Si nuclei.

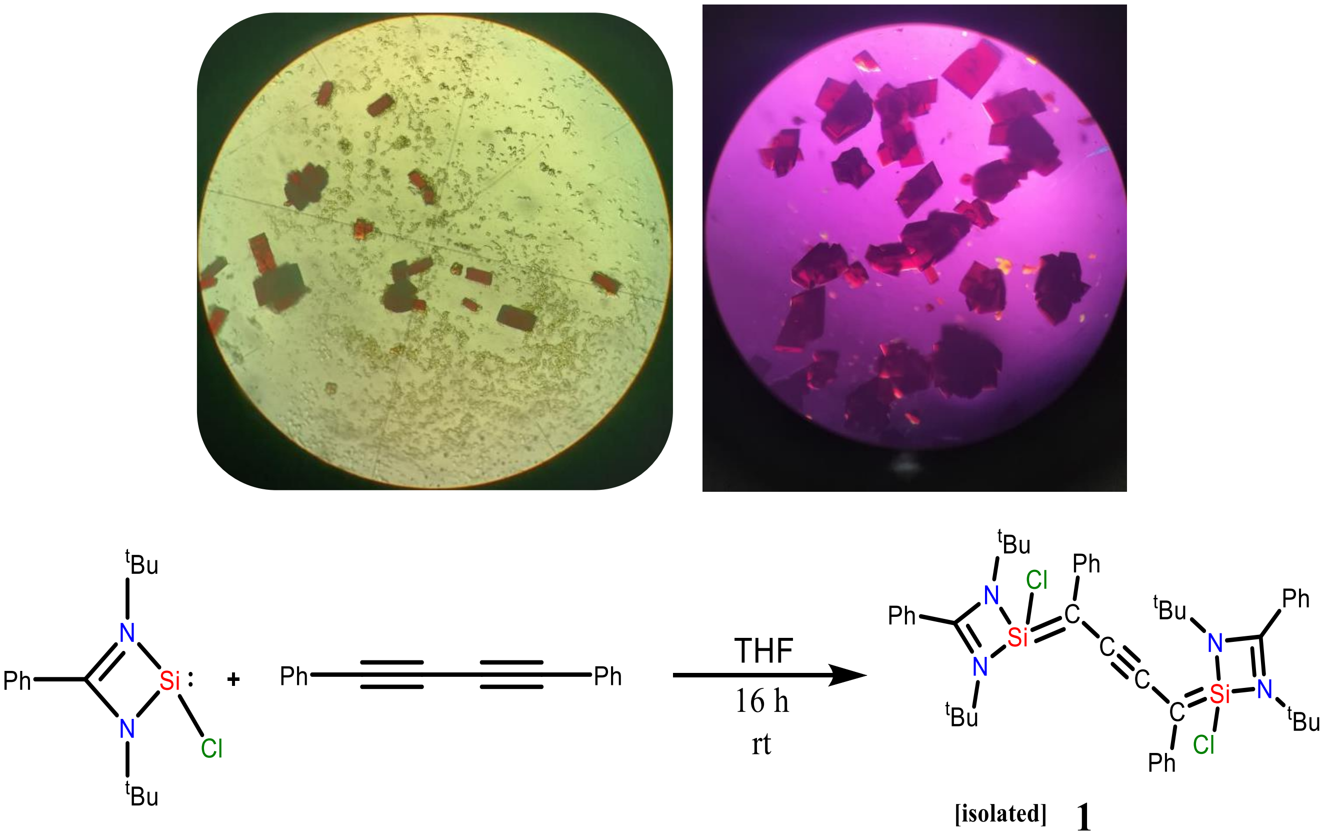

[isolated] **1**

Scheme S1. Synthesis of compound **1** in THF. Picture of the single crystals of **1** on the glass plate submerged in fluorinated paratone oil.

1.3. Synthesis of compound 2

Amidinato-chlorosilylene, L(Cl)Si: (L = PhC(N^{t}Bu)$_2$) and 1,4-diphenylbuta-1,3-diyne were reacted at a 3:1 molar ratio in toluene at room temperature and stirred for 24 h to obtain a red colored reaction mixture (**Scheme 1**). The reaction mixture was filtered, and the resultant filtrate was dried under vacuum. The dried mass was extracted with diethyl ether. The ether solution was concentrated to about 8 mL and stored at room temperature for crystallization. The single crystals of (L)SiCl$_2$(Ph$_2$C$_4$)SiCl$_2$(L) (**2**) were isolated after 15 days in 22% yield. It is observed from the mass spectrometric data that the reaction proceeds with the formation of compound **1** as well and then converts to **2**. Compound **2** was characterized by SC-XRD, NMR, and mass spectrometry.

2. **Spectroscopic data**

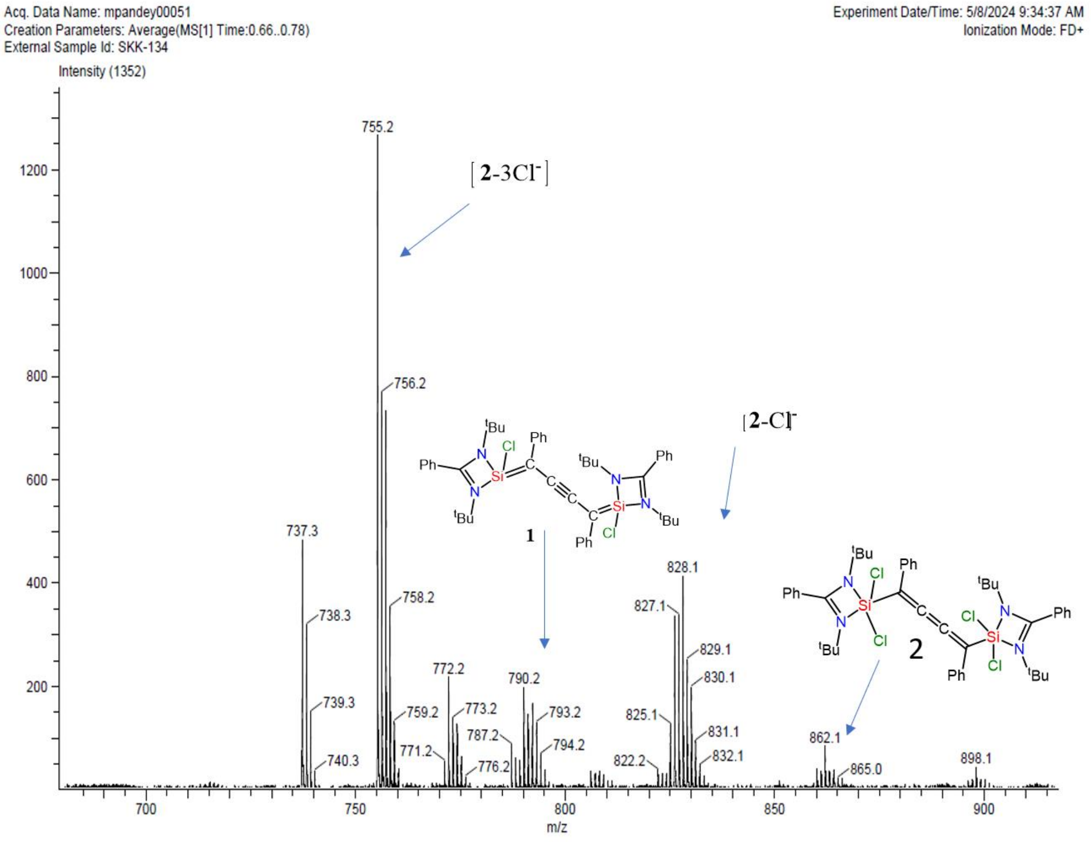

Figure S1. Mass spectrum displaying mass peak of **1**, **2**, and fragmentation peaks of **2**. The sample was dissolved in toluene for mass spectrometric measurements. The mass peak of **2** is comparatively less intense due to its fragmentation. This measurement was carried out from toluene reaction solution.

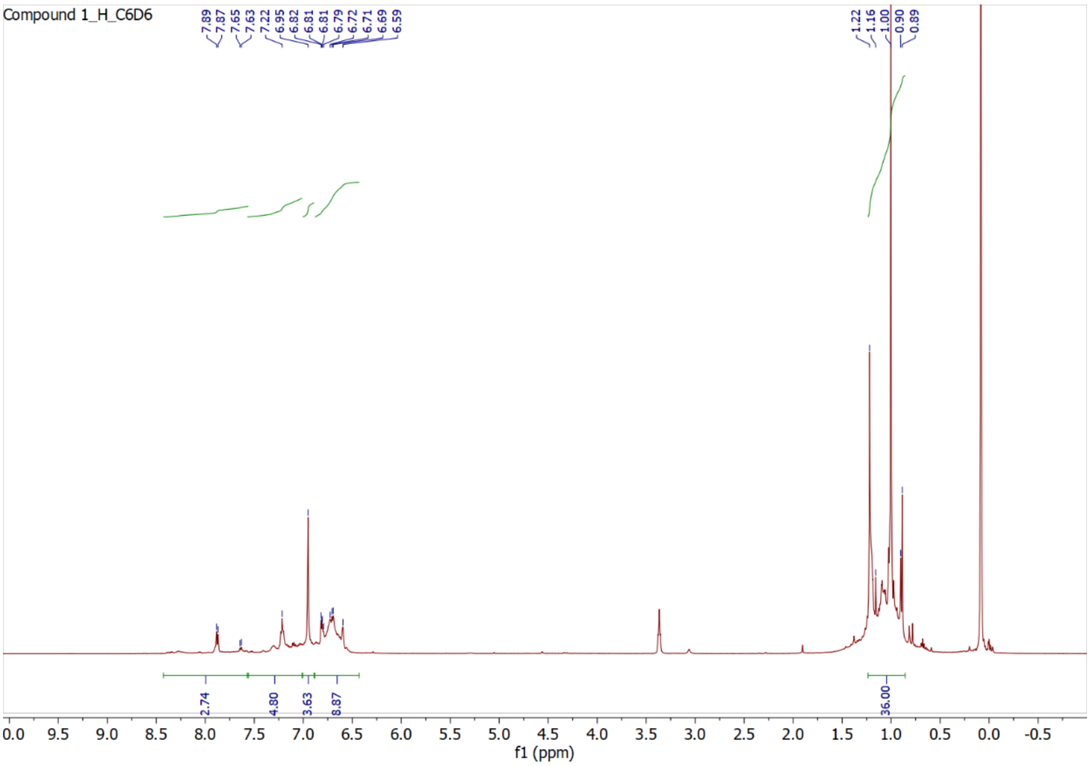

Figure S2. ^{1}H NMR of compounds **1** (recorded in C_6D_6).

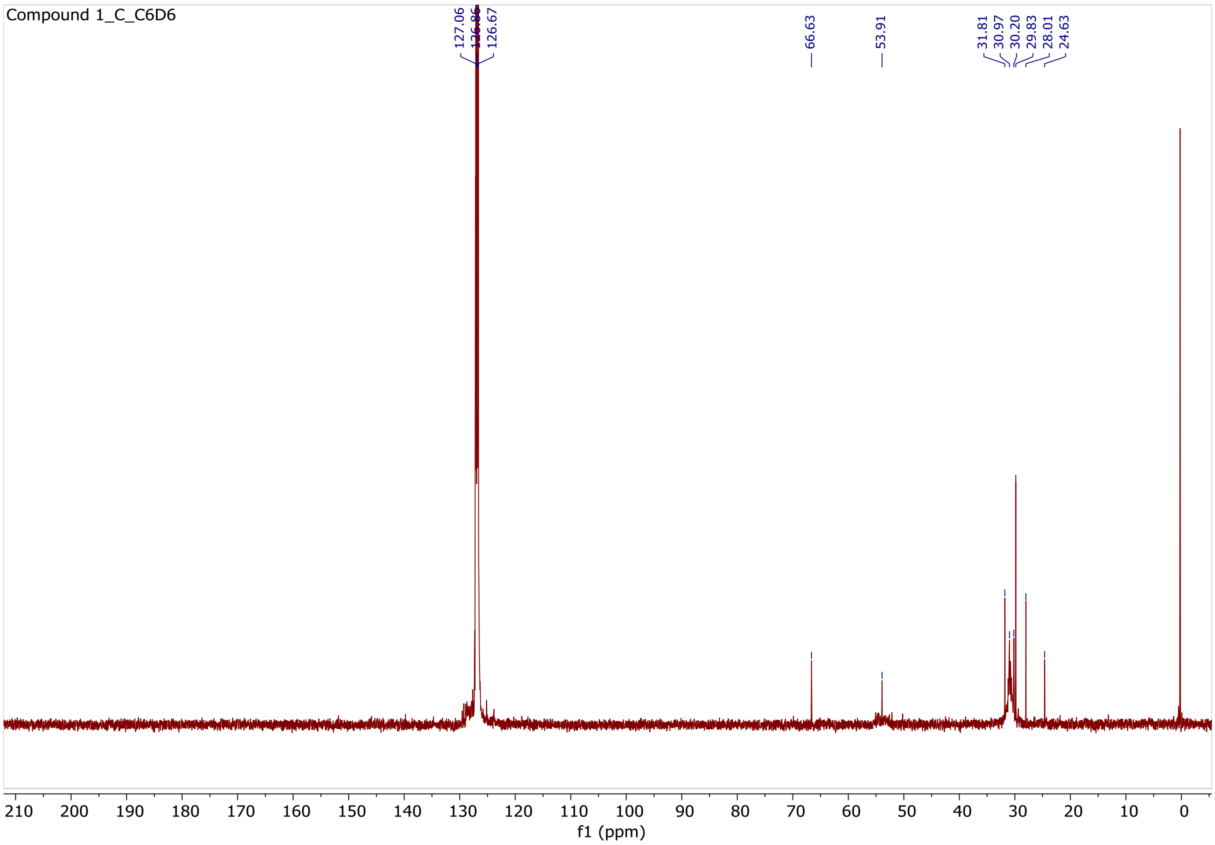

Figure S3. ^{13}C NMR of compounds **1** (recorded in C_6D_6).

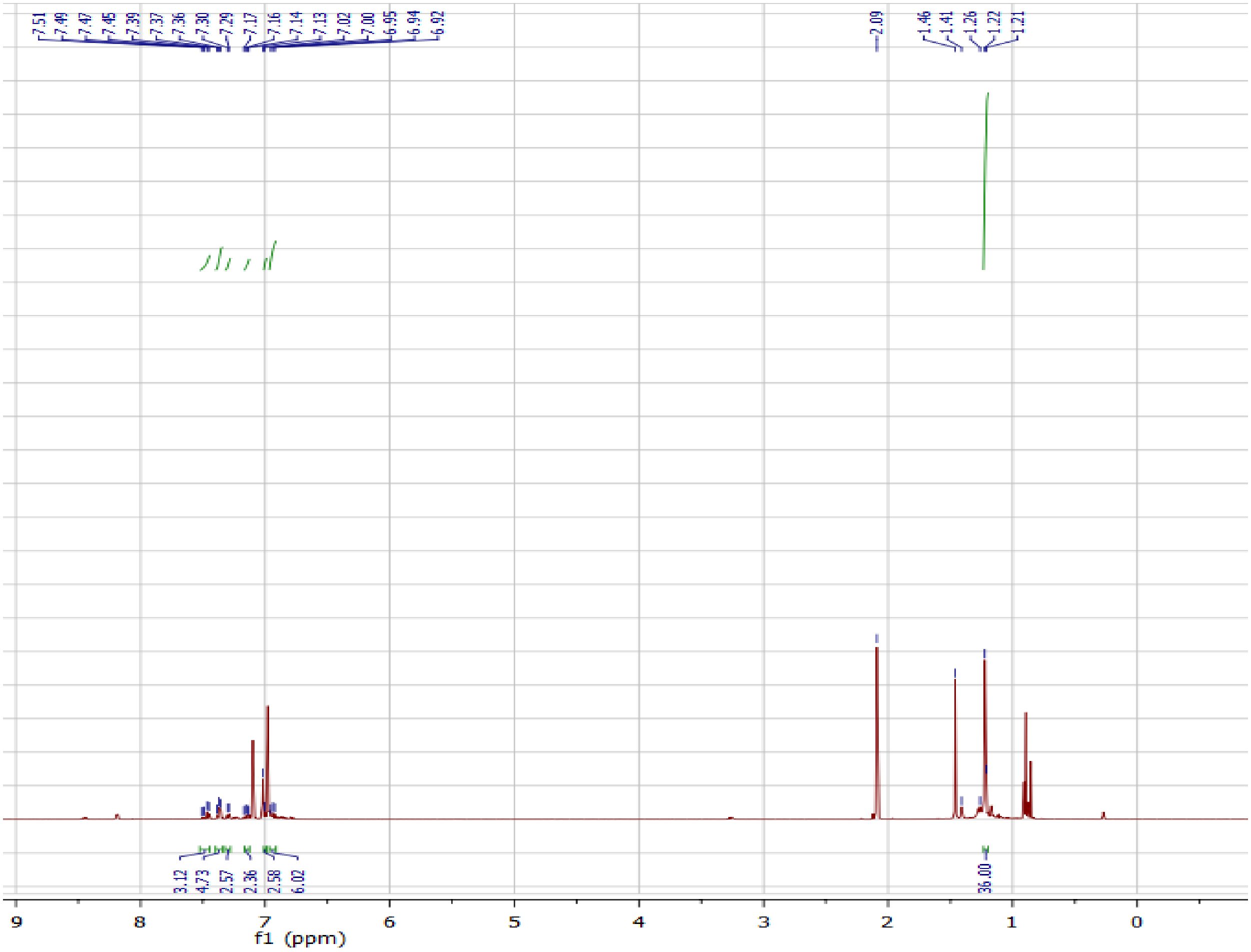

Figure S4. ^{1}H NMR of compound **2** (recorded in Toluene-D_8).

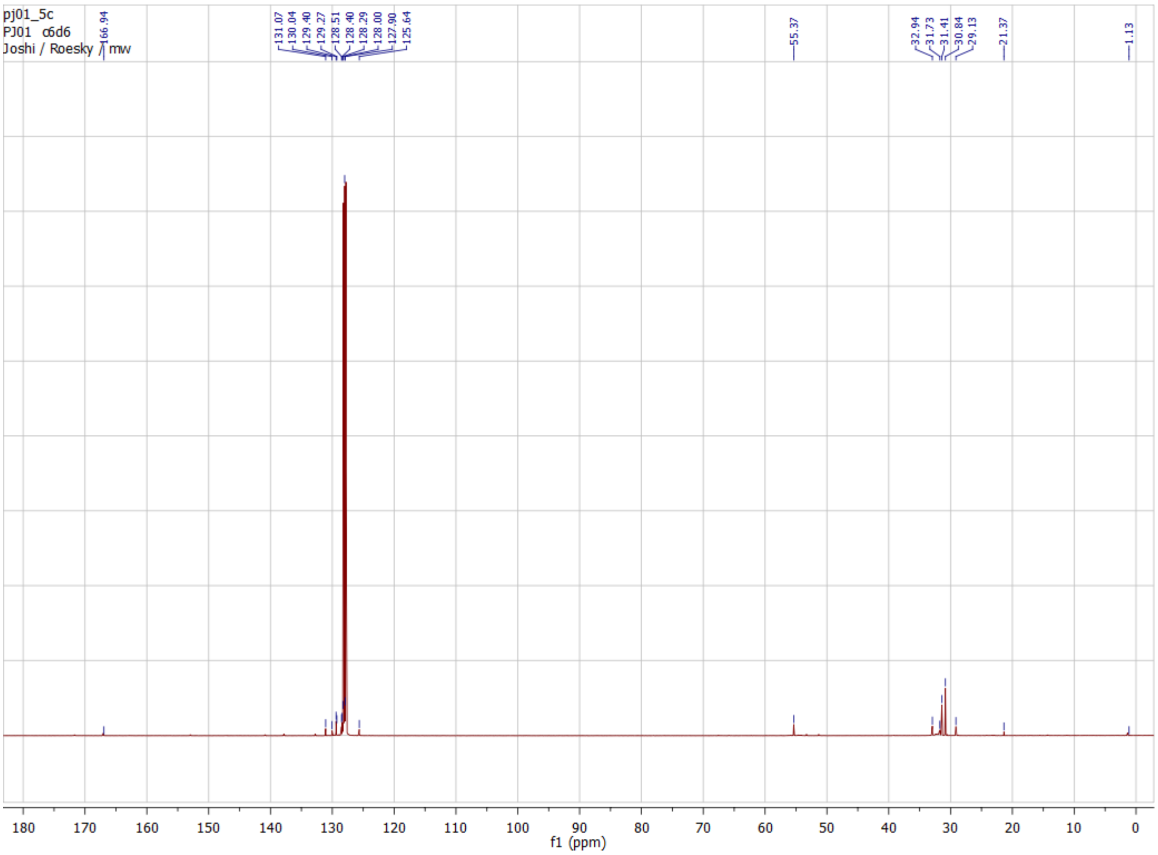

Figure S5. ^{13}C NMR of compound **2** (recorded in C_6D_6).

.

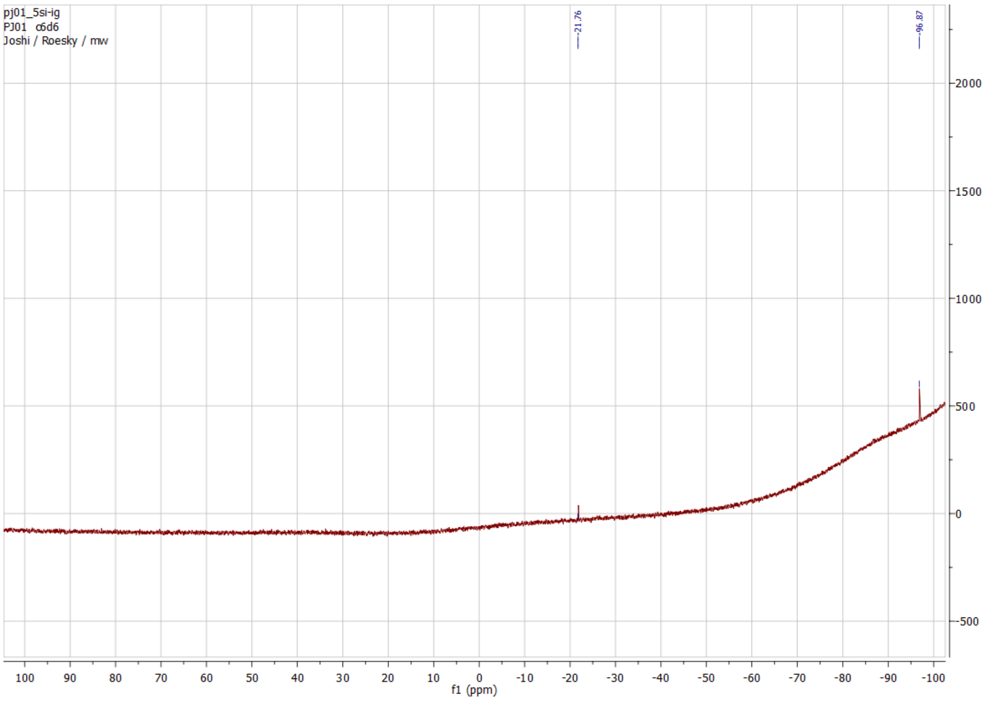

Figure S6. ^{29}Si NMR of compounds **2** in C_6D_6.

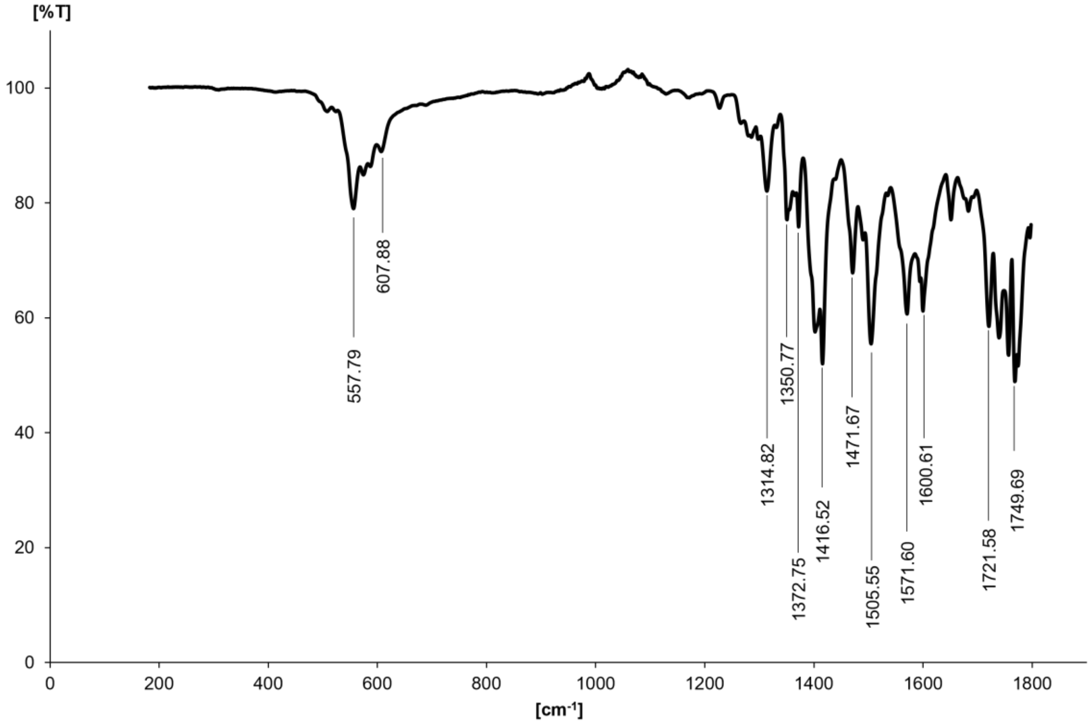

Figure S7. Solid state FT-IR spectra of **1** recorded using KBr pellet.

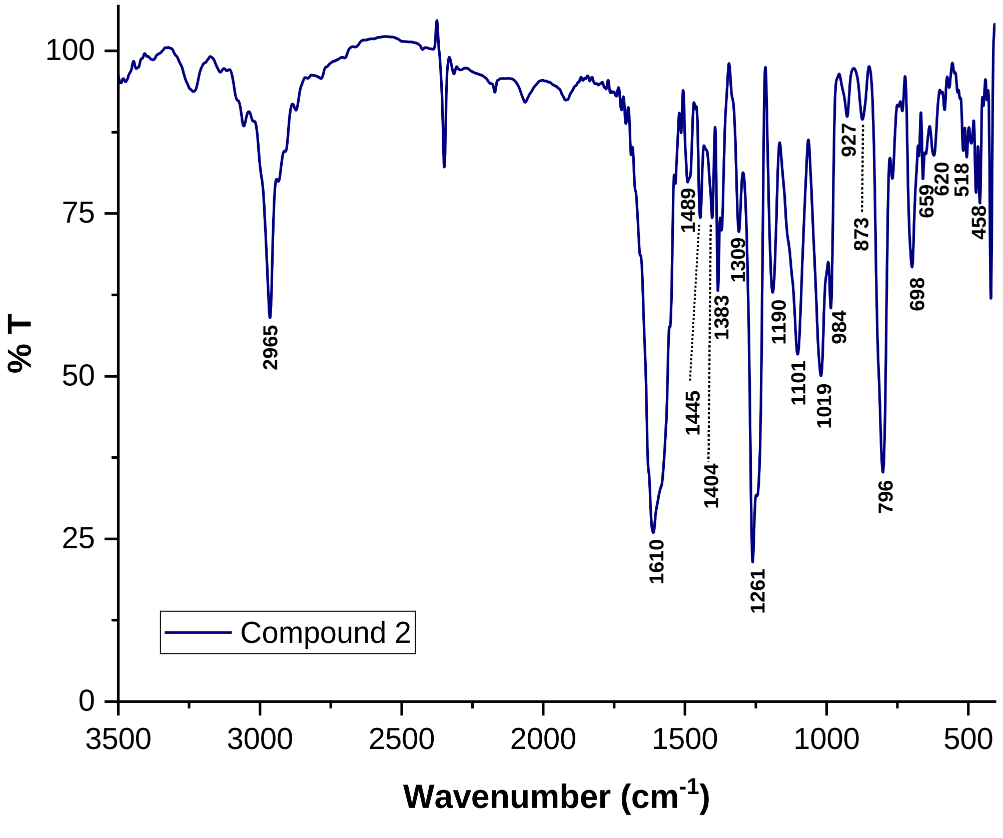

Figure S8. Solid state FT-IR spectra of **2** recorded using KBr pellet.

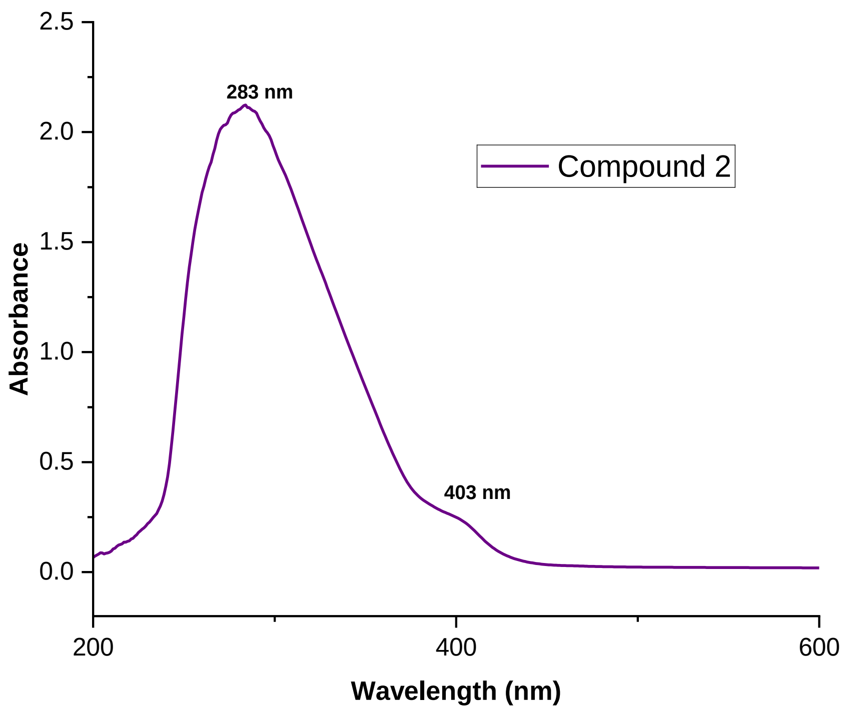

Figure S9. UV-VIS spectrum of **1** recorded in THF.

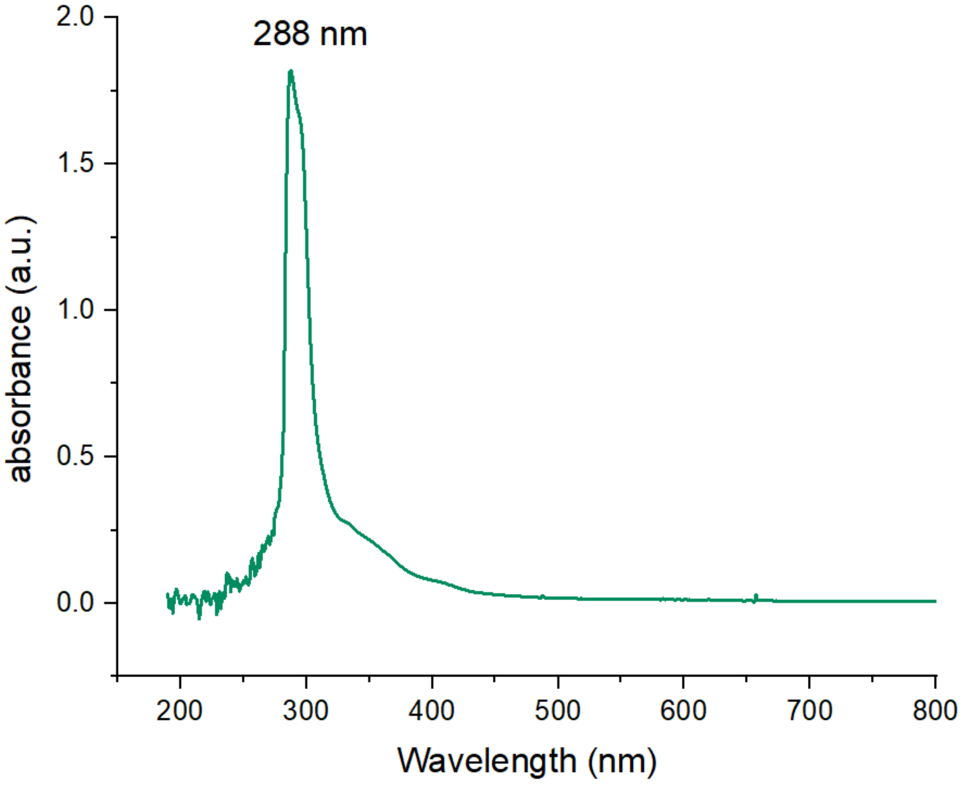

Figure 10. UV-VIS spectra of **2** recorded in toluene.

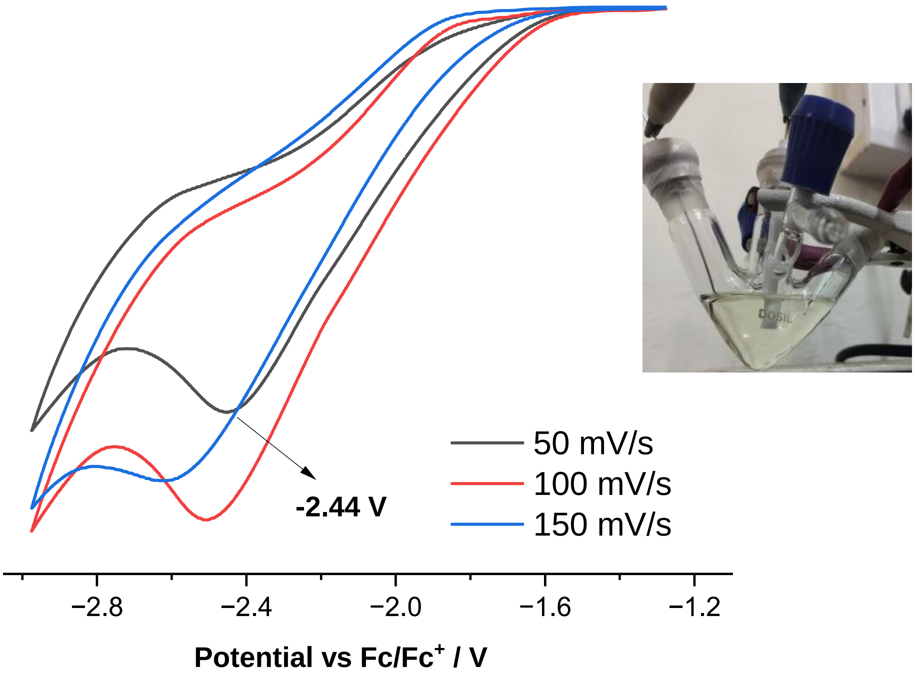

Figure S11. Cyclic voltammogram of (L)Si-Cl + Ph_2C_4 (1:3) in 0.1 M THF solution of [*n*-Bu_4N]PF_6 with RE: Ag, WE: GC, and CE: Pt.

Amidinato-chlorosilylene was added in the same electrochemical cell after the CV measurement 1,4-diphenylbuta-1,3-diyne was completed in 1:3 molar ratio. (light yellow solution). This electrochemical study reveals an irreversible reduction process at -2.44 V.

3. Crystallographic data

An orange-red block-shaped crystal of **1** and a colourless block-shaped crystal of **1** were mounted on a nylon loop with perfluoroether oil. The datasets were collected using a Bruker D8 three-circle diffractometer, equipped with a Bruker Photon III C7 CMOS detector and an INCOATEC microfocus source (Mo K_α radiation) with INCOATEC Quazar mirror optics. All data were integrated with SAINT.[20] A Multi-Scan absorption correction using SADABS 2016/2 was applied.[21] The structure was solved by SHELXT and refined by full-matrix least-squares methods against F^2 using SHELXL-2019/3.[22-23] All non-hydrogen atoms were refined with anisotropic displacement parameters. All hydrogen atoms were refined isotropic on calculated positions using a riding model with their U_{iso} values constrained to 1.5 times the U_{eq} of their pivot atoms for terminal sp^3 carbon atoms and 1.2 times for all other carbon atoms. Crystallographic data for the structures reported in this paper have been deposited with the Cambridge Crystallographic Data Centre.[24] These data can be obtained free of charge from The Cambridge Crystallographic Data Centre via http://www.ccdc.cam.ac.uk. This report and the CIF file were generated using FinalCif.[25]

Table S1. Crystallographic table for compound **1** and **2**.

Compound	**1**	**2**
CCDC	2503170	2400672
Empirical formula	$C_{46}H_{56}Cl_2N_4Si_2$	$C_{46}H_{56}Cl_4N_4Si_2$
Formula weight	792.02	862.92
Temperature (K)	100(2)	100(2)
Wavelength (Å)	0.71073	0.71073
Crystal system	Triclinic	Monoclinic
Space group	*P*-1	$P2_1/c$
a (Å)	10.695(2)	10.373(2)
b (Å)	12.850(2)	18.725(4)
c (Å)	17.050(3)	12.465(3)
α (deg)	69.50(2)	90
β (deg)	81.06(2)	109.86(2)
γ (deg)	81.83(2)	90
V (Å^3)	2158.4(7)	2277.1(9)
Z	2	2
Density (Mg/m^3)	1.219	1.259
μ (mm^{-1})	0.243	0.349
Crystal size (mm)	0.26 × 0.13 × 0.10	0.253 x 0.083 x 0.074
Crystal colour, shape	Orange red, block	Colourless block
Θ range (deg)	1.936 to 28.705	2.049 to 26.388
Reflections collected	91603	176882
Independent reflections	11096	4660
R_{int}	0.0379	0.0285
Data/restraints/parameters	11096 / 111 / 539	4660 / 0 /259
$R_1(I > 2\sigma(I))$	0.0381	0.0267
wR_2(all data)	0.1044	0.0663
$\Delta\rho_{max}/\Delta\rho min$ (e Å^{-3})	0.503 and -0.257	0.380 and -0.221

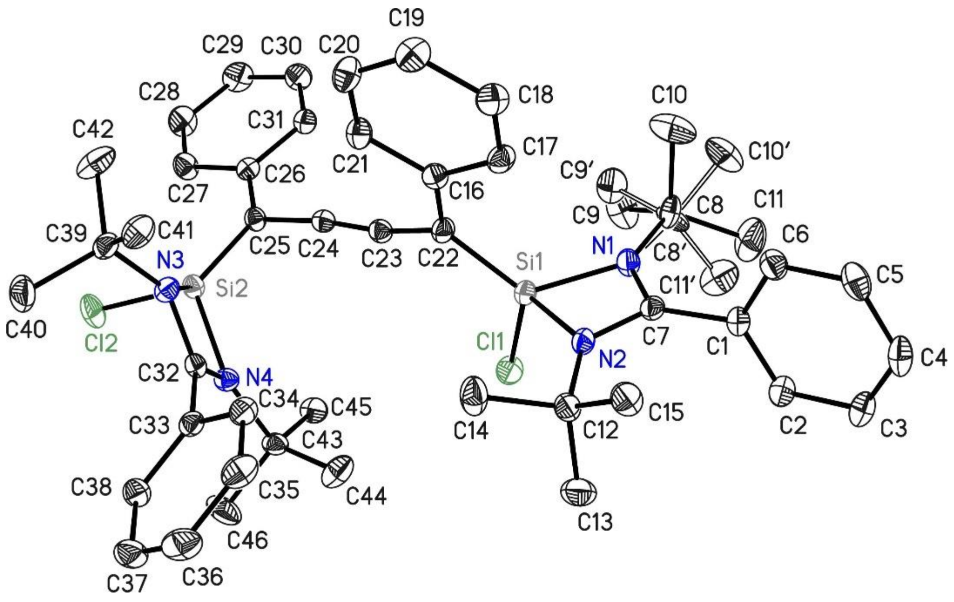

Figure S12. The asymmetric unit of **1** with thermal ellipsoids at 50% probability level. The hydrogen atoms are omitted for clarity. The disordered tBu group was refined with distance restraints and restraints for the anisotropic displacement parameters. The occupancy of the minor component refined to 0.456(5).

Table S2. Bond lengths [Å] and angles [°] of **1**.

Cl(1)-Si(1)	2.0329(7)	C(1)-C(7)	1.4850(17)
Cl(2)-Si(2)	2.0720(6)	C(2)-C(3)	1.3928(18)
Si(1)-C(22)	1.7582(13)	C(3)-C(4)	1.384(2)
Si(1)-N(2)	1.8097(11)	C(4)-C(5)	1.388(2)
Si(1)-N(1)	1.8205(12)	C(5)-C(6)	1.3878(19)
Si(1)-C(7)	2.2712(13)	C(7)-N(1)	1.3354(17)
Si(2)-C(25)	1.7447(13)	N(1)-C(8)	1.465(8)
Si(2)-N(4)	1.8145(11)	N(1)-C(8')	1.506(8)
Si(2)-N(3)	1.8241(11)	C(8)-C(11)	1.515(7)
Si(2)-C(32)	2.2684(13)	C(8)-C(10)	1.529(8)
N(2)-C(7)	1.3443(16)	C(8)-C(9)	1.538(8)
N(2)-C(12)	1.4860(16)	C(8')-C(9')	1.511(9)
N(3)-C(32)	1.3429(16)	C(8')-C(10')	1.516(9)
N(3)-C(39)	1.4827(16)	C(8')-C(11')	1.520(9)
N(4)-C(32)	1.3323(16)	C(12)-C(13)	1.528(2)
N(4)-C(43)	1.4871(16)	C(12)-C(14)	1.5296(19)
C(1)-C(2)	1.3913(18)	C(12)-C(15)	1.5303(18)
C(1)-C(6)	1.3931(18)	C(16)-C(21)	1.4129(18)

C(16)-C(17)	1.4151(17)	Cl(1)-Si(1)-C(7)	109.38(4)
C(16)-C(22)	1.4598(18)	C(25)-Si(2)-N(4)	123.14(6)
C(17)-C(18)	1.3863(19)	C(25)-Si(2)-N(3)	122.24(6)
C(18)-C(19)	1.390(2)	N(4)-Si(2)-N(3)	72.18(5)
C(19)-C(20)	1.387(2)	C(25)-Si(2)-Cl(2)	119.84(5)
C(20)-C(21)	1.382(2)	N(4)-Si(2)-Cl(2)	104.71(4)
C(22)-C(23)	1.4227(17)	N(3)-Si(2)-Cl(2)	104.54(4)
C(23)-C(24)	1.2190(18)	C(25)-Si(2)-C(32)	133.80(5)
C(24)-C(25)	1.4284(16)	N(4)-Si(2)-C(32)	35.96(5)
C(25)-C(26)	1.4600(17)	N(3)-Si(2)-C(32)	36.30(5)
C(26)-C(31)	1.4069(18)	Cl(2)-Si(2)-C(32)	106.36(4)
C(26)-C(27)	1.4088(17)	C(7)-N(2)-C(12)	132.31(11)
C(27)-C(28)	1.3853(19)	C(7)-N(2)-Si(1)	90.90(8)
C(28)-C(29)	1.388(2)	C(12)-N(2)-Si(1)	136.07(8)
C(29)-C(30)	1.384(2)	C(32)-N(3)-C(39)	129.57(11)
C(30)-C(31)	1.3910(18)	C(32)-N(3)-Si(2)	90.18(8)
C(32)-C(33)	1.4750(17)	C(39)-N(3)-Si(2)	135.71(8)
C(33)-C(34)	1.3910(18)	C(32)-N(4)-C(43)	131.15(11)
C(33)-C(38)	1.3963(18)	C(32)-N(4)-Si(2)	90.93(8)
C(34)-C(35)	1.3872(19)	C(43)-N(4)-Si(2)	137.51(9)
C(35)-C(36)	1.386(2)	C(2)-C(1)-C(6)	120.50(12)
C(36)-C(37)	1.388(2)	C(2)-C(1)-C(7)	120.44(11)
C(37)-C(38)	1.3876(19)	C(6)-C(1)-C(7)	118.85(11)
C(39)-C(42)	1.5237(19)	C(1)-C(2)-C(3)	119.39(12)
C(39)-C(40)	1.5245(19)	C(4)-C(3)-C(2)	120.15(13)
C(39)-C(41)	1.5318(19)	C(3)-C(4)-C(5)	120.27(13)
C(43)-C(46)	1.5205(18)	C(6)-C(5)-C(4)	120.10(13)
C(43)-C(45)	1.5218(18)	C(5)-C(6)-C(1)	119.54(13)
C(43)-C(44)	1.5268(19)	N(1)-C(7)-N(2)	106.02(11)
		N(1)-C(7)-C(1)	128.88(11)
C(22)-Si(1)-N(2)	121.26(6)	N(2)-C(7)-C(1)	125.07(11)
C(22)-Si(1)-N(1)	126.52(6)	N(1)-C(7)-Si(1)	53.27(6)
N(2)-Si(1)-N(1)	72.26(5)	N(2)-C(7)-Si(1)	52.81(6)
C(22)-Si(1)-Cl(1)	115.82(5)	C(1)-C(7)-Si(1)	177.73(9)
N(2)-Si(1)-Cl(1)	108.76(4)	C(7)-N(1)-C(8)	132.8(3)
N(1)-Si(1)-Cl(1)	104.24(5)	C(7)-N(1)-C(8')	132.4(4)
C(22)-Si(1)-C(7)	134.80(5)	C(7)-N(1)-Si(1)	90.72(8)
N(2)-Si(1)-C(7)	36.28(5)	C(8)-N(1)-Si(1)	136.4(3)
N(1)-Si(1)-C(7)	36.01(5)	C(8')-N(1)-Si(1)	136.3(4)

N(1)-C(8)-C(11)	114.1(5)
N(1)-C(8)-C(10)	107.0(5)
C(11)-C(8)-C(10)	111.4(5)
N(1)-C(8)-C(9)	107.2(5)
C(11)-C(8)-C(9)	108.6(6)
C(10)-C(8)-C(9)	108.2(5)
N(1)-C(8')-C(9')	105.2(6)
N(1)-C(8')-C(10')	110.4(6)
C(9')-C(8')-C(10')	111.3(6)
N(1)-C(8')-C(11')	106.2(5)
C(9')-C(8')-C(11')	111.3(7)
C(10')-C(8')-C(11')	112.0(6)
N(2)-C(12)-C(13)	108.41(11)
N(2)-C(12)-C(14)	106.08(10)
C(13)-C(12)-C(14)	109.78(12)
N(2)-C(12)-C(15)	112.60(11)
C(13)-C(12)-C(15)	111.52(11)
C(14)-C(12)-C(15)	108.29(12)
C(21)-C(16)-C(17)	115.95(12)
C(21)-C(16)-C(22)	119.83(11)
C(17)-C(16)-C(22)	124.20(11)
C(18)-C(17)-C(16)	121.84(12)
C(17)-C(18)-C(19)	120.90(12)
C(20)-C(19)-C(18)	118.24(13)
C(21)-C(20)-C(19)	121.48(13)
C(20)-C(21)-C(16)	121.59(12)
C(23)-C(22)-C(16)	117.87(11)
C(23)-C(22)-Si(1)	119.04(9)
C(16)-C(22)-Si(1)	123.06(9)
C(24)-C(23)-C(22)	167.87(13)
C(23)-C(24)-C(25)	172.41(13)
C(24)-C(25)-C(26)	121.64(11)
C(24)-C(25)-Si(2)	104.99(9)
C(26)-C(25)-Si(2)	133.35(9)
C(31)-C(26)-C(27)	116.27(11)
C(31)-C(26)-C(25)	120.47(11)
C(27)-C(26)-C(25)	123.26(11)
C(28)-C(27)-C(26)	121.69(13)
C(27)-C(28)-C(29)	120.97(13)
C(30)-C(29)-C(28)	118.53(13)
C(29)-C(30)-C(31)	120.85(13)
C(30)-C(31)-C(26)	121.69(12)
N(4)-C(32)-N(3)	106.49(11)
N(4)-C(32)-C(33)	126.98(11)
N(3)-C(32)-C(33)	126.53(11)
N(4)-C(32)-Si(2)	53.11(6)
N(3)-C(32)-Si(2)	53.53(6)
C(33)-C(32)-Si(2)	175.94(9)
C(34)-C(33)-C(38)	120.60(12)
C(34)-C(33)-C(32)	119.86(11)
C(38)-C(33)-C(32)	119.55(11)
C(35)-C(34)-C(33)	119.20(12)
C(36)-C(35)-C(34)	120.37(13)
C(35)-C(36)-C(37)	120.44(13)
C(38)-C(37)-C(36)	119.74(13)
C(37)-C(38)-C(33)	119.64(13)
N(3)-C(39)-C(42)	105.24(11)
N(3)-C(39)-C(40)	109.69(11)
C(42)-C(39)-C(40)	111.02(12)
N(3)-C(39)-C(41)	111.89(10)
C(42)-C(39)-C(41)	108.20(12)
C(40)-C(39)-C(41)	110.67(12)
N(4)-C(43)-C(46)	109.84(11)
N(4)-C(43)-C(45)	105.26(10)
C(46)-C(43)-C(45)	109.64(12)
N(4)-C(43)-C(44)	111.36(11)
C(46)-C(43)-C(44)	111.02(12)
C(45)-C(43)-C(44)	109.56(11)

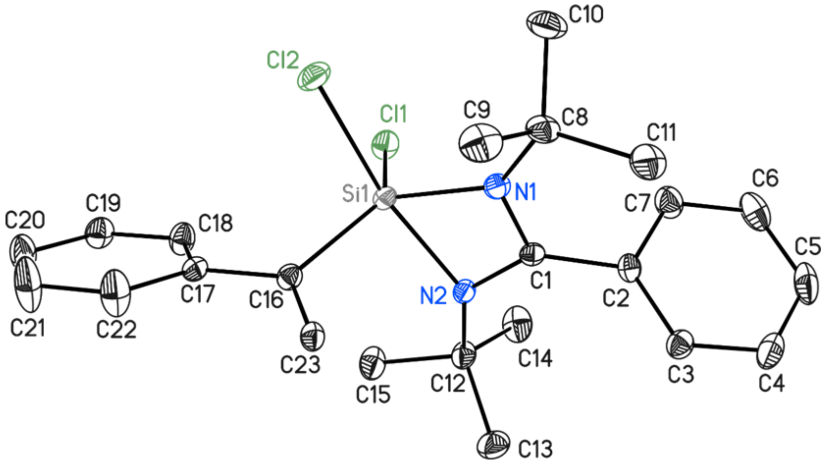

Figure S13. The asymmetric unit of **2** with thermal ellipsoids at 50% probability level. The hydrogen atoms are omitted for clarity. The asymmetric unit contains a half of the molecule **2**.

Table S3. Bond lengths [Å] and angles [°] of **2**.

Cl(1)-Si(1)	2.1045(6)	C(12)-C(15)	1.5303(17)
C(2)-C(3)	1.3900(17)	C(16)-C(23)	1.3333(17)
C(2)-C(7)	1.3944(18)	C(16)-C(17)	1.4872(16)
C(2)-C(1)	1.4900(17)	C(17)-C(22)	1.3918(19)
N(2)-C(1)	1.3092(15)	C(17)-C(18)	1.3974(17)
N(2)-C(12)	1.4876(15)	C(18)-C(19)	1.3896(18)
N(2)-Si(1)	1.9470(11)	C(19)-C(20)	1.3779(19)
N(1)-C(1)	1.3674(16)	C(20)-C(21)	1.385(2)
N(1)-C(8)	1.5010(15)	C(21)-C(22)	1.388(2)
N(1)-Si(1)	1.8016(11)	C(23)-C(23)#1	1.265(2)
C(1)-Si(1)	2.3308(13)		
Si(1)-C(16)	1.8893(13)	C(3)-C(2)-C(7)	119.58(12)
Si(1)-Cl(2)	2.1843(6)	C(3)-C(2)-C(1)	123.47(11)
C(3)-C(4)	1.3901(19)	C(7)-C(2)-C(1)	116.95(11)
C(4)-C(5)	1.386(2)	C(1)-N(2)-C(12)	130.14(10)
C(5)-C(6)	1.381(2)	C(1)-N(2)-Si(1)	89.19(7)
C(6)-C(7)	1.389(2)	C(12)-N(2)-Si(1)	138.23(8)
C(8)-C(10)	1.5290(18)	C(1)-N(1)-C(8)	129.86(10)
C(8)-C(11)	1.5328(19)	C(1)-N(1)-Si(1)	93.69(7)
C(8)-C(9)	1.537(2)	C(8)-N(1)-Si(1)	136.08(8)
C(12)-C(13)	1.5281(18)	N(2)-C(1)-N(1)	106.51(10)
C(12)-C(14)	1.5302(18)	N(2)-C(1)-C(2)	127.37(11)

N(1)-C(1)-C(2)	125.35(11)	N(1)-C(8)-C(10)	109.09(10)
N(2)-C(1)-Si(1)	56.64(6)	N(1)-C(8)-C(11)	113.28(10)
N(1)-C(1)-Si(1)	50.48(6)	C(10)-C(8)-C(11)	107.83(11)
C(2)-C(1)-Si(1)	164.85(8)	N(1)-C(8)-C(9)	107.48(10)
N(1)-Si(1)-C(16)	125.09(5)	C(10)-C(8)-C(9)	112.09(12)
N(1)-Si(1)-N(2)	69.69(5)	C(11)-C(8)-C(9)	107.13(11)
C(16)-Si(1)-N(2)	96.57(5)	N(2)-C(12)-C(13)	109.40(10)
N(1)-Si(1)-Cl(1)	110.80(4)	N(2)-C(12)-C(14)	111.47(10)
C(16)-Si(1)-Cl(1)	122.36(4)	C(13)-C(12)-C(14)	111.58(11)
N(2)-Si(1)-Cl(1)	89.62(4)	N(2)-C(12)-C(15)	107.53(10)
N(1)-Si(1)-Cl(2)	100.24(4)	C(13)-C(12)-C(15)	107.85(11)
C(16)-Si(1)-Cl(2)	91.16(4)	C(14)-C(12)-C(15)	108.87(11)
N(2)-Si(1)-Cl(2)	169.67(3)	C(23)-C(16)-C(17)	122.28(11)
Cl(1)-Si(1)-Cl(2)	92.03(2)	C(23)-C(16)-Si(1)	113.61(9)
N(1)-Si(1)-C(1)	35.84(4)	C(17)-C(16)-Si(1)	123.56(8)
C(16)-Si(1)-C(1)	117.63(5)	C(22)-C(17)-C(18)	117.66(11)
N(2)-Si(1)-C(1)	34.17(4)	C(22)-C(17)-C(16)	121.02(11)
Cl(1)-Si(1)-C(1)	98.61(4)	C(18)-C(17)-C(16)	121.31(11)
Cl(2)-Si(1)-C(1)	135.56(4)	C(19)-C(18)-C(17)	121.09(12)
C(2)-C(3)-C(4)	119.97(12)	C(20)-C(19)-C(18)	120.45(12)
C(5)-C(4)-C(3)	120.15(13)	C(19)-C(20)-C(21)	119.20(13)
C(6)-C(5)-C(4)	120.10(13)	C(20)-C(21)-C(22)	120.49(13)
C(5)-C(6)-C(7)	120.10(13)	C(21)-C(22)-C(17)	121.09(13)
C(6)-C(7)-C(2)	120.06(13)	C(23)#1-C(23)-C(16)	175.10(17)

Symmetry transformations used to generate equivalent atoms:

#1 -x+1,-y+1,-z+1

4. Computational Methods

Geometry optimizations and vibrational frequencies calculations of $(LSi)_2$-C_4Ph_2 (**2**) and $(LSi)_2$-$(SiMe_3)_2C_4$ (**3**) compounds in singlet electronic state have been carried out at the PBE0-D3(BJ)/Def2TZVPP level in the gas phase.[1] All the calculations were carried out using the Gaussian 16 program package.[2] The absence of imaginary frequencies assures the minima on the potential energy surface. We have performed NBO [3] calculation using the NBO 6.0[4] program to estimate natural bond orbitals, partial charges, Wiberg bond indices (WBI)[5a], and AIM analysis using the AIMALL package.[5b-5c] The nature of Si–C bonds in compounds **2** and **3** were analyzed by energy decomposition analysis (EDA)[7] coupled with natural orbital for chemical valence (NOCV)[8] using the ADF 2018.105 program package.[9] EDA-NOCV calculations were carried out at the PBE0-D3(BJ)/TZ2P[10] level using the geometries optimized at PBE0-D3(BJ)/def2-TZVPP level. The EDA-NOCV method involves the decomposition of the intrinsic interaction energy (ΔE_{int}) between two fragments into four energy components as follows:

$$\Delta E_{\text{int}} = \Delta E_{\text{elstat}} + \Delta E_{\text{Pauli}} + \Delta E_{\text{orb}} + \Delta E_{\text{disp}} \qquad (1)$$

where the electrostatic ΔE_{elstat} term originates from the quasi-classical electrostatic interaction between the unperturbed charge distributions of the prepared fragments, the Pauli repulsion ΔE_{Pauli} (repulsion energy due to the interactions of same spins between the fragments) is the energy change associated with the transformation from the superposition of the unperturbed electron densities of the isolated fragments to the wavefunction, which properly obeys the Pauli principle through explicit anti-symmetrization and renormalization of the production of the wavefunction. Dispersion interaction, ΔE_{disp} (equivalent to attractive forces due to instantaneous fluctuation of electron clouds in the fragment before and after the bond formation) is also obtained as we used D3(BJ). The orbital term ΔE_{orb} comes from (constructive interference during spatial mixing of orbitals of the fragments) the mixing of orbitals, charge transfer and polarization between the isolated fragments. This can be further divided into contributions from each irreducible representation of the point group of an interacting system as follows:

$$\Delta E_{orb} = \sum_{r} \Delta E_{r} \qquad (2)$$

The combined EDA-NOCV method is able to partition the total orbital interactions into pairwise contributions of the orbital interactions, which are important in providing a complete picture of the bonding. The charge deformation $\Delta\rho_k(r)$, which comes from the mixing of the orbital pairs $\psi_k(r)$ and $\psi_{-k}(r)$ of the interacting fragments, gives the magnitude and the shape of the charge flow due to the orbital interactions (Equation 3), and the associated orbital energy ΔE_{orb} presents the amount of orbital energy coming from such interaction (Equation 4).

$$\Delta\rho_{orb}(r) = \sum_{k}\Delta\rho_{k}(r) = \sum_{k=1}^{N/2}\nu_{k}[-\psi_{-k}^{2}(r) + \psi_{k}^{2}(r)] \qquad (3)$$

$$\Delta E_{Orb} = \sum_{k}\Delta E_{Orb}^{k} = \sum_{k}\nu_{k}\left[-F_{-k,-k}^{TS} + F_{k,k}^{TS}\right] \qquad (4)$$

Readers are further referred to the recent review articles to know more about the EDA-NOCV method and its application. [11] Also, a very recent report related to equation (1) has been critically discussed by different researchers. [12]

…………………

Analysis of IR spectrum of **1**:

The infrared (IR) spectrum of the compound **1** shows a strong absorption band at 2965 cm^{-1} is attributed to the aliphatic C–H stretching vibrations[13] originating from the tert-butyl groups and other alkyl moieties present in the molecule. The aromatic C=C stretching vibrations linked to the phenyl rings are evidenced by the distinct peak at 1610 cm^{-1}, which can also overlap with C=N stretching vibrations in conjugated systems, supported further by the bands in the ranges 1489-1404 cm^{-1}, which represent aromatic ring stretches and C–H bending modes.[14-15] The methyl symmetric bending vibration from the tert-butyl groups is observed clearly at 1383 cm^{-1}.[16] The absorption near 1261 cm^{-1} is assignable to the C–N stretching vibrations with contributions likely overlapping with Si–N and Si–C stretches observed in the range 1190-1019 cm^{-1}, characteristic of organosilicon compounds.[17] Out-of-plane aromatic C–H bending vibrations appear prominently between 927-796 cm^{-1} and again at lower frequencies near 698-620 cm^{-1}, confirming the substituted phenyl rings in the structure.[18] The presence of chlorine bound to silicon atoms is clearly indicated by a low-frequency stretching vibration in the range 518-458 cm^{-1}, attributed to Si–Cl bonds.[19]

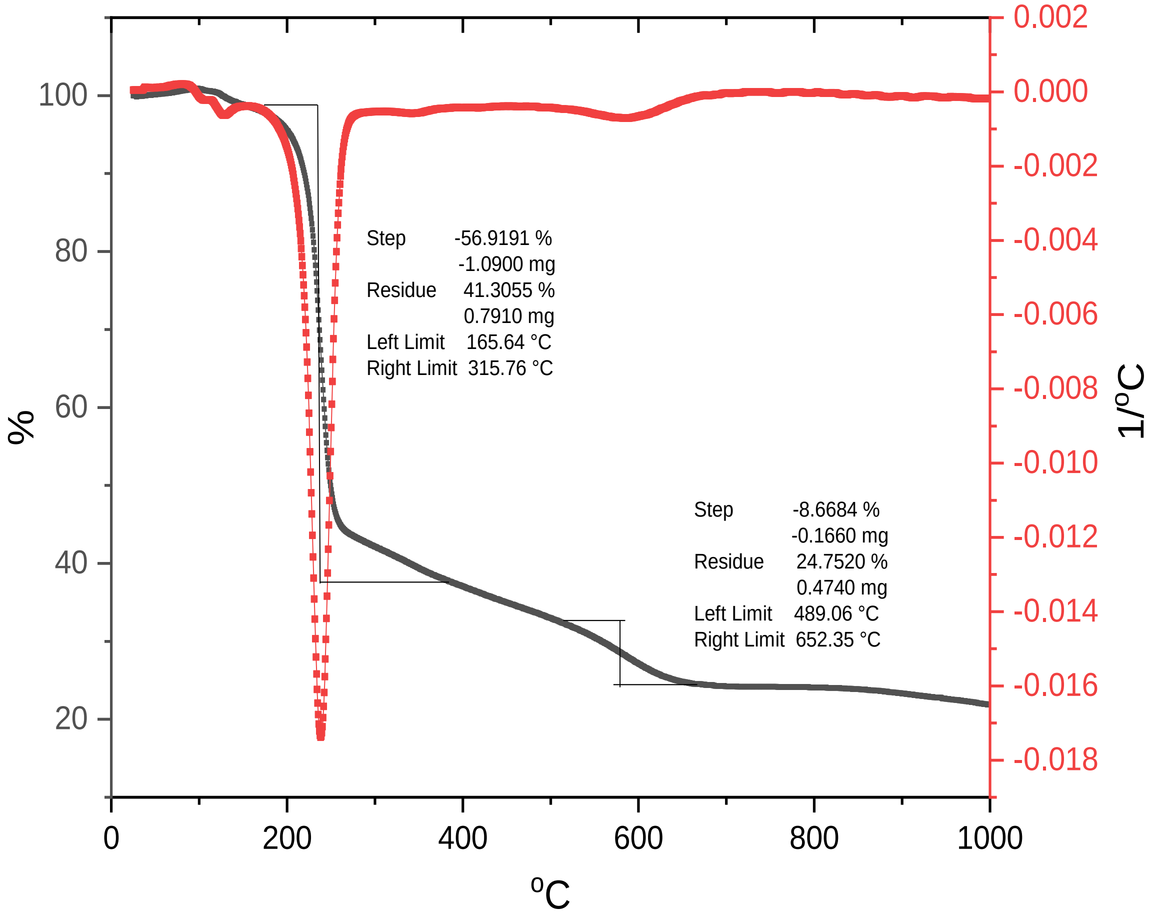

Figure S14. Thermogravimetric analysis plot of compound **1**.

…………………

Optimized coordinates

Compound **2**

Cl	5.438907000	7.204867000	2.146033000
C	0.885062000	6.592897000	2.337280000
N	2.778037000	8.160438000	2.681336000
N	2.801547000	6.450914000	3.974821000
C	2.102019000	7.096931000	3.011195000
Si	4.239314000	7.550394000	3.822872000
Cl	5.612704000	6.512436000	5.151432000
C	-0.381235000	7.092455000	2.605860000
H	-0.510542000	7.874627000	3.341871000
C	-1.482576000	6.579401000	1.938954000
H	-2.469332000	6.967836000	2.158902000
C	-1.321838000	5.576960000	0.994338000
H	-2.183915000	5.179738000	0.472970000
C	-0.053993000	5.085961000	0.715809000
H	0.078284000	4.307086000	-0.024788000
C	1.047246000	5.588611000	1.387761000
H	2.039805000	5.207238000	1.179693000
C	2.428083000	5.295695000	4.826378000
C	2.600223000	5.702084000	6.291049000
H	1.915752000	6.515639000	6.535745000
H	3.614651000	6.019008000	6.512965000
H	2.358050000	4.852237000	6.931721000
C	3.311932000	4.105165000	4.463979000
H	3.139656000	3.812715000	3.425933000
H	3.065258000	3.254761000	5.102634000
H	4.366915000	4.337083000	4.588738000
C	0.971834000	4.869919000	4.669365000
H	0.746408000	4.455501000	3.689687000
H	0.284216000	5.687417000	4.877962000
H	0.786086000	4.091078000	5.410115000
C	2.534091000	9.169809000	1.643120000
C	1.220464000	9.904028000	1.906978000
H	1.156036000	10.774695000	1.251884000
H	1.178826000	10.251375000	2.941020000
H	0.354695000	9.275788000	1.708252000
C	2.528914000	8.543646000	0.250611000
H	2.438116000	9.329905000	-0.501212000
H	1.692622000	7.857710000	0.120493000

H	3.459322000	8.003238000	0.074011000
C	3.662265000	10.192906000	1.728514000
H	3.531532000	10.933548000	0.938592000
H	4.639074000	9.728343000	1.602876000
H	3.645703000	10.711405000	2.685902000
C	4.405680000	9.169147000	4.765756000
C	5.573289000	10.058293000	4.695070000
C	6.876539000	9.599599000	4.486611000
H	7.066456000	8.537760000	4.418291000
C	7.929074000	10.496242000	4.395516000
H	8.934162000	10.119918000	4.246409000
C	7.706136000	11.863229000	4.483143000
H	8.532067000	12.558746000	4.397701000
C	6.412949000	12.331562000	4.669219000
H	6.221356000	13.396610000	4.723260000
C	5.362890000	11.438672000	4.779532000
H	4.350150000	11.794902000	4.918749000
C	3.364452000	9.473898000	5.526949000
Cl	1.265946000	11.315168000	10.424839000
C	5.672234000	11.625534000	9.007857000
N	3.542108000	10.367939000	8.987646000
N	3.387655000	12.339470000	8.158372000
C	4.236027000	11.432069000	8.708270000
Si	1.928024000	11.286598000	8.427228000
Cl	0.336842000	12.587939000	7.763650000
C	6.652653000	11.276988000	8.089260000
H	6.374274000	10.866150000	7.128182000
C	7.989015000	11.464522000	8.404470000
H	8.746460000	11.194197000	7.679568000
C	8.349442000	11.993614000	9.634554000
H	9.394857000	12.139205000	9.877692000
C	7.368951000	12.332513000	10.556683000
H	7.644788000	12.741722000	11.520684000
C	6.032198000	12.148069000	10.245678000
H	5.261759000	12.412720000	10.960058000
C	3.593957000	13.772514000	7.841967000
C	3.008814000	14.064041000	6.460756000
H	3.583732000	13.547459000	5.691995000
H	1.969309000	13.762360000	6.381732000
H	3.072469000	15.135864000	6.264502000
C	2.903933000	14.612728000	8.915456000
H	3.341378000	14.405496000	9.894302000
H	3.037547000	15.674666000	8.699405000
H	1.837230000	14.401493000	8.961982000
C	5.062459000	14.181574000	7.783122000
H	5.551461000	14.165444000	8.754059000
H	5.633218000	13.559946000	7.095263000
H	5.094029000	15.208064000	7.414069000
C	3.946043000	9.077803000	9.565676000
C	5.036209000	8.410723000	8.731081000
H	5.194693000	7.393557000	9.094240000
H	4.744960000	8.352230000	7.682213000
H	5.984852000	8.939487000	8.800865000
C	4.412065000	9.262185000	11.008907000
H	4.595084000	8.284280000	11.458217000
H	5.337730000	9.832960000	11.062156000
H	3.645692000	9.773253000	11.593113000
C	2.719682000	8.169444000	9.567095000
H	2.975811000	7.231057000	10.060417000
H	1.889189000	8.622437000	10.108088000
H	2.395107000	7.940378000	8.554133000
C	1.445143000	9.925845000	7.193608000
C	0.175268000	9.195377000	7.199390000
C	-0.732481000	9.312301000	8.254171000
H	-0.509686000	9.966463000	9.085685000
C	-1.921739000	8.602742000	8.249895000
H	-2.608653000	8.710090000	9.080572000
C	-2.235793000	7.765190000	7.191437000
H	-3.167827000	7.213381000	7.187244000
C	-1.346102000	7.643908000	6.131152000
H	-1.588352000	6.993959000	5.298304000
C	-0.158555000	8.349658000	6.135414000
H	0.540084000	8.263233000	5.310901000
C	2.387635000	9.681926000	6.294036000

Compound **3**

Cl	8.559382000	3.494829000	6.097647000
Si	9.409649000	4.344571000	4.408602000
O	11.095628000	4.310019000	5.086558000
N	7.859211000	4.001354000	3.298213000
N	9.832137000	3.170564000	3.093693000
C	8.582299000	3.196569000	2.568989000
C	9.426860000	6.192729000	4.195826000
S	11.710606000	4.576401000	6.448518000
C	6.423023000	4.325365000	3.238023000
C	10.917603000	2.169186000	2.916385000
C	8.128902000	2.523176000	1.333272000
Si	10.038535000	7.143734000	2.676769000
C	8.966323000	6.919387000	5.190711000
O	12.161387000	3.366270000	7.049844000
O	10.978945000	5.530864000	7.213806000
C	13.232291000	5.456551000	5.891136000
C	5.915343000	4.580732000	1.821275000
C	6.190457000	5.582634000	4.064588000
C	5.639630000	3.173849000	3.869773000
C	12.237861000	2.881094000	2.641534000
C	11.008222000	1.331343000	4.192582000
C	10.679844000	1.211364000	1.754664000
C	8.410455000	3.121046000	0.110699000
C	7.391329000	1.347836000	1.377459000
C	10.968195000	8.658099000	3.238290000
C	8.565618000	7.647985000	1.642423000
C	11.179923000	6.081824000	1.640332000
C	8.509690000	7.638275000	6.130255000
F	13.999924000	4.661981000	5.155183000
F	13.917667000	5.846436000	6.956630000
F	12.920059000	6.527194000	5.169362000
H	6.528721000	5.323213000	1.311343000
H	4.901744000	4.979140000	1.888414000
H	5.879127000	3.678684000	1.213932000
H	6.563339000	5.465647000	5.081260000
H	5.118427000	5.775789000	4.118757000
H	6.666839000	6.454907000	3.623929000
H	5.735424000	2.260582000	3.282684000
H	4.580732000	3.434785000	3.914639000
H	5.992467000	2.981042000	4.882494000
H	12.212723000	3.374057000	1.669535000
H	13.043432000	2.145170000	2.628750000
H	12.468672000	3.619315000	3.401361000
H	11.270724000	1.926976000	5.063018000
H	11.773273000	0.562580000	4.069045000
H	10.055379000	0.835341000	4.388012000
H	9.809160000	0.575859000	1.903896000
H	11.554369000	0.562342000	1.693293000
H	10.579294000	1.724689000	0.799780000

H	8.980792000	4.041629000	0.081818000
C	7.948130000	2.547990000	-1.062004000
H	7.185300000	0.876165000	2.329962000
C	6.935635000	0.773704000	0.201867000
H	10.328095000	9.297904000	3.844911000
H	11.297988000	9.234975000	2.370347000
H	11.847008000	8.391107000	3.826061000
H	8.048942000	6.779961000	1.229625000
H	8.895525000	8.268837000	0.805070000
H	7.852462000	8.223689000	2.234781000
H	12.078220000	5.804697000	2.193641000
H	11.490423000	6.645051000	0.756472000
H	10.700098000	5.162397000	1.303081000
C	8.049672000	8.365294000	7.125078000
C	7.209655000	1.374205000	-1.018261000
H	8.163105000	3.021083000	-2.012013000
H	6.364222000	-0.145347000	0.239693000
Si	8.067090000	10.213320000	6.911049000
Si	7.437487000	7.416399000	8.645112000
H	6.847938000	0.926852000	-1.935715000
Cl	8.913912000	11.059369000	5.218608000
O	6.379069000	10.248310000	6.237973000
N	9.620239000	10.556538000	8.018373000
N	7.648016000	11.388507000	8.225865000
C	8.898547000	11.361173000	8.749043000
C	6.517175000	5.895665000	8.085828000
C	8.909646000	6.923665000	9.686228000
C	6.288425000	8.477216000	9.674062000
S	5.758946000	9.978384000	4.879103000
C	11.056273000	10.231504000	8.076832000
C	6.564998000	12.392700000	8.403067000
C	9.352965000	12.031787000	9.985880000
H	7.161699000	5.257808000	7.481884000
H	6.188834000	5.319228000	8.954608000
H	5.638015000	6.156505000	7.495795000
H	9.420686000	7.796334000	10.096263000
H	8.580321000	6.305305000	10.525643000
H	9.627220000	6.348487000	9.098704000
H	5.392514000	8.752192000	9.115832000
H	5.974371000	7.914687000	10.557108000
H	6.765143000	9.397828000	10.012485000
O	5.303276000	11.186733000	4.277887000
O	6.489335000	9.024216000	4.112269000
C	4.241317000	9.095912000	5.443813000
C	11.566658000	9.977073000	9.492783000
C	11.286931000	8.973397000	7.251092000
C	11.838828000	11.382225000	7.442549000
C	5.243669000	11.684674000	8.682512000
C	6.473441000	13.227667000	7.125018000
C	6.807784000	13.353091000	9.561614000
C	9.068933000	11.432280000	11.207091000
C	10.093637000	13.205263000	9.944501000
F	3.475401000	9.889606000	6.182516000
F	3.552017000	8.703898000	4.381643000
F	4.558707000	8.026429000	6.165059000
H	10.954905000	9.234262000	10.004137000
H	12.580371000	9.579274000	9.423695000
H	11.603601000	10.879277000	10.099823000
H	10.912297000	9.089503000	6.235005000
H	12.358796000	8.779871000	7.195180000
H	10.811008000	8.101734000	7.693473000
H	11.744086000	12.296133000	8.028844000
H	12.897620000	11.121149000	7.396091000
H	11.484210000	11.574085000	6.430258000
H	5.269821000	11.193319000	9.655305000
H	4.440001000	12.422668000	8.695947000
H	5.009159000	10.945691000	7.924571000
H	6.207151000	12.630650000	6.256701000
H	5.710690000	13.998699000	7.248622000
H	7.427069000	13.720729000	6.926026000
H	7.680020000	13.985614000	9.408662000
H	5.935296000	14.004860000	9.622797000
H	6.908798000	12.842358000	10.517825000
H	8.496052000	10.513198000	11.233699000
C	9.531989000	12.001787000	12.381240000
H	10.301681000	13.678233000	8.993086000
C	10.549939000	13.775924000	11.121530000
C	10.273538000	13.173729000	12.340298000
H	9.315148000	11.527383000	13.330171000
H	11.123754000	14.693566000	11.085919000
H	10.635788000	13.618342000	13.25887300

LSi

Cl	0.929931000	11.804415000	10.416856000
C	5.730107000	11.658879000	9.039772000
N	3.518186000	10.487273000	9.346117000
N	3.384807000	12.510714000	8.671100000
C	4.249140000	11.560685000	9.035877000
Si	1.969050000	11.261135000	8.587091000
C	6.464220000	11.323426000	7.900150000
H	5.948911000	10.990202000	7.003960000
C	7.853826000	11.417104000	7.915008000
H	8.420110000	11.155156000	7.026336000
C	8.512973000	11.845209000	9.065659000
H	9.596408000	11.917576000	9.075951000
C	7.780503000	12.180560000	10.203948000
H	8.290398000	12.514548000	11.102498000
C	6.392291000	12.088257000	10.193031000
H	5.817024000	12.347421000	11.076938000
C	3.586634000	13.854460000	8.121756000
C	4.156140000	13.779777000	6.701591000
H	5.163355000	13.354244000	6.701581000
H	3.513590000	13.162215000	6.065941000
H	4.212578000	14.782512000	6.265094000
C	2.199955000	14.498394000	8.082884000
H	1.773120000	14.561498000	9.087964000
H	2.266321000	15.506600000	7.664233000
H	1.516191000	13.915832000	7.454607000
C	4.500616000	14.688374000	9.021885000
H	4.131381000	14.683136000	10.051998000
H	5.528163000	14.318494000	9.015096000
H	4.513751000	15.723183000	8.665375000
C	3.898462000	9.114296000	9.690134000
C	4.476313000	8.391865000	8.469044000
H	4.669190000	7.340794000	8.708394000
H	3.769823000	8.431998000	7.633847000
H	5.419944000	8.844865000	8.152810000
C	4.896451000	9.088257000	10.849489000
H	5.039438000	8.054618000	11.179970000
H	5.871453000	9.485300000	10.559035000
H	4.516837000	9.670828000	11.694481000
C	2.605404000	8.424723000	10.127790000
H	2.804970000	7.379897000	10.382082000
H	2.175644000	8.924444000	11.000632000
H	1.862264000	8.440398000	9.322064000

Ph2-C4

C	4.518625000	10.353229000	5.364711000
C	5.795818000	10.832994000	4.972797000
C	6.636114000	10.041931000	4.169066000
H	6.297360000	9.060789000	3.852002000
C	7.885297000	10.516447000	3.788078000
H	8.527562000	9.898818000	3.167345000
C	8.313943000	11.778597000	4.199452000
H	9.290957000	12.145586000	3.899461000
C	7.485798000	12.568701000	4.996842000
H	7.816505000	13.551668000	5.318850000
C	6.234612000	12.104369000	5.383812000
H	5.586150000	12.714813000	6.004125000
C	3.419944000	9.939960000	5.701394000
C	1.097865000	9.066601000	6.413198000
C	-0.179344000	8.587031000	6.805297000
C	-1.018773000	9.377656000	7.610368000
H	-0.679337000	10.358289000	7.928278000
C	-2.267961000	8.903353000	7.991601000
H	-2.909546000	9.520637000	8.613379000
C	-2.697470000	7.641848000	7.579150000
H	-3.674480000	7.275015000	7.879347000
C	-1.870194000	6.852187000	6.780420000
H	-2.201583000	5.869728000	6.457565000
C	-0.619010000	7.316311000	6.393194000
H	0.028778000	6.706204000	5.771846000
C	2.196513000	9.479892000	6.076433000

TS-1

C	4.338756000	10.522651000	5.212192000
C	5.660319000	10.801881000	4.781568000
C	6.644801000	9.797851000	4.843990000
H	6.368846000	8.805198000	5.185535000
C	7.955112000	10.084019000	4.481778000
H	8.709456000	9.304644000	4.534527000
C	8.301631000	11.366206000	4.053905000
H	9.327638000	11.586736000	3.774910000
C	7.329051000	12.363544000	3.980729000
H	7.595925000	13.360940000	3.644686000
C	6.014868000	12.088282000	4.337936000
H	5.255542000	12.861685000	4.285407000
C	3.230747000	10.259877000	5.656098000
C	5.594822000	11.618867000	9.120005000
N	3.637930000	10.159133000	8.606746000
N	3.296974000	12.277887000	8.299721000
C	4.228516000	11.329111000	8.615598000
Si	1.840184000	11.229671000	8.750228000
Cl	0.141502000	12.543046000	7.908371000
C	6.728018000	11.287164000	8.377585000
H	6.618204000	10.853265000	7.388896000
C	7.996841000	11.548632000	8.889067000
H	8.874540000	11.299684000	8.299759000
C	8.138304000	12.130523000	10.147329000
H	9.128327000	12.329734000	10.546900000
C	7.005271000	12.461859000	10.891589000
H	7.110396000	12.916622000	11.872188000
C	5.737386000	12.214932000	10.376658000
H	4.850740000	12.475459000	10.948383000
C	3.498217000	13.677733000	7.847400000
C	2.871528000	13.825161000	6.454960000
H	3.374340000	13.158513000	5.748928000
H	1.809819000	13.581708000	6.461685000
H	2.990812000	14.856778000	6.107263000
C	2.832525000	14.620051000	8.852668000
H	3.305207000	14.517592000	9.835364000
H	2.944098000	15.658509000	8.523565000
H	1.766682000	14.403579000	8.949489000
C	4.970808000	14.073030000	7.704982000
H	5.500948000	14.122328000	8.656924000
H	5.512002000	13.397174000	7.036927000
H	4.995455000	15.071702000	7.258048000
C	4.086515000	8.879064000	9.168699000
C	5.069209000	8.218545000	8.196291000
H	5.312232000	7.207992000	8.541510000
H	4.625570000	8.150519000	7.198763000
H	6.000030000	8.786688000	8.127437000
C	4.713326000	8.995379000	10.562441000
H	4.850819000	7.992872000	10.981544000
H	5.688122000	9.486391000	10.541826000
H	4.054128000	9.556625000	11.232988000
C	2.835871000	8.000298000	9.271404000
H	3.109648000	7.004192000	9.631997000
H	2.111772000	8.432891000	9.968523000
H	2.352939000	7.896833000	8.296221000
C	0.994121000	9.712732000	6.830261000
C	-0.305288000	9.170602000	7.084596000
C	-0.845108000	9.083623000	8.374824000
H	-0.274486000	9.473794000	9.212453000
C	-2.099247000	8.517951000	8.568536000
H	-2.510571000	8.458553000	9.571645000
C	-2.828937000	8.035893000	7.482745000
H	-3.810353000	7.597539000	7.638207000
C	-2.298942000	8.122095000	6.194999000
H	-2.865320000	7.752574000	5.345321000
C	-1.045477000	8.684887000	5.991762000
H	-0.627731000	8.759801000	4.992813000
C	2.037293000	9.973264000	6.222273000

TS-2

C	4.280989000	10.538490000	4.738083000
C	5.507764000	11.253862000	4.780909000
C	6.780032000	10.645214000	4.816817000
H	6.839205000	9.562870000	4.800289000
C	7.935877000	11.417686000	4.875754000
H	8.900826000	10.918244000	4.909992000
C	7.871064000	12.810833000	4.894990000
H	8.777493000	13.406890000	4.942712000
C	6.618190000	13.426816000	4.833927000
H	6.543972000	14.511320000	4.831241000
C	5.457235000	12.668964000	4.768589000
H	4.488756000	13.156301000	4.702098000
C	3.163708000	10.277872000	5.270398000
C	5.285300000	11.355433000	9.125197000
N	3.684593000	9.528253000	8.442036000
N	2.883998000	11.517517000	8.399576000
C	3.997542000	10.817667000	8.644018000
Si	1.960198000	9.976832000	8.165028000
Cl	0.941479000	9.650015000	9.949818000
C	6.323461000	11.630312000	8.234809000
H	6.197661000	11.452046000	7.172396000
C	7.516639000	12.158249000	8.719549000
H	8.317299000	12.377570000	8.020316000

C	7.673790000	12.406662000	10.081511000
H	8.606581000	12.819019000	10.454431000
C	6.633911000	12.126740000	10.967953000
H	6.754363000	12.315015000	12.030339000
C	5.435732000	11.603224000	10.493073000
H	4.625839000	11.373846000	11.179412000
C	2.554424000	12.948375000	8.493456000
C	1.318809000	13.136981000	7.612641000
H	1.536730000	12.865181000	6.575583000
H	0.482957000	12.519627000	7.962067000
H	0.997982000	14.181609000	7.644638000
C	2.226506000	13.307058000	9.944705000
H	3.104208000	13.181786000	10.584904000
H	1.909309000	14.352800000	10.004737000
H	1.418844000	12.677895000	10.329641000
C	3.695745000	13.815971000	7.967449000
H	4.558765000	13.810852000	8.636209000
H	4.020661000	13.478175000	6.979982000
H	3.342650000	14.847997000	7.882897000
C	4.472735000	8.288793000	8.541075000
C	5.544304000	8.269936000	7.451440000
H	6.048773000	7.298701000	7.446874000
H	5.100789000	8.441513000	6.466554000
H	6.299191000	9.040708000	7.625334000
C	5.096626000	8.150178000	9.929941000
H	5.577376000	7.170774000	10.013536000
H	5.856784000	8.913842000	10.109609000
H	4.329366000	8.225601000	10.707001000
C	3.471782000	7.156870000	8.312452000
H	3.989070000	6.194156000	8.328817000
H	2.707000000	7.144186000	9.096857000
H	2.979178000	7.260533000	7.340914000
C	1.108255000	9.644393000	6.614021000
C	-0.318757000	9.281871000	6.498472000
C	-0.876017000	8.235917000	7.244865000
H	-0.241587000	7.662495000	7.915974000
C	-2.224222000	7.910602000	7.119967000
H	-2.635125000	7.087483000	7.698039000
C	-3.043863000	8.633712000	6.254335000
H	-4.096929000	8.383985000	6.161703000
C	-2.502100000	9.683834000	5.512825000
H	-3.135935000	10.262698000	4.845624000
C	-1.152669000	10.002608000	5.632583000
H	-0.723770000	10.822125000	5.061315000
C	1.917657000	9.918464000	5.558843000
C	1.210235000	8.084853000	1.887983000
N	3.226193000	7.378213000	3.215766000
N	3.547613000	9.024241000	1.889102000
C	2.624810000	8.169152000	2.324478000
Si	4.831798000	8.352184000	3.103891000
Cl	5.846484000	7.022562000	1.629451000
C	0.189823000	8.596645000	2.691673000
C	-1.134156000	8.487710000	2.276503000
C	-1.442696000	7.867724000	1.066427000
H	-2.477785000	7.780508000	0.748573000
C	-0.422951000	7.354353000	0.266275000
H	-0.658441000	6.866741000	-0.675090000
C	0.903690000	7.463388000	0.674346000
H	1.703152000	7.061151000	0.058685000
C	3.432409000	10.250522000	1.094781000
C	4.824723000	10.883107000	1.105473000
H	5.125290000	11.149498000	2.123347000
H	5.565543000	10.191572000	0.689951000
H	4.826206000	11.793569000	0.498889000
C	3.048230000	9.915655000	-0.349544000
H	2.030260000	9.523984000	-0.413327000
H	3.100217000	10.820870000	-0.963669000
H	3.737944000	9.173513000	-0.763544000
C	2.421474000	11.220745000	1.711785000
H	1.404860000	10.821364000	1.662286000
H	2.439778000	12.170363000	1.165944000
C	2.811567000	6.115238000	3.827870000
C	1.565514000	6.292532000	4.700042000
H	1.389798000	5.381355000	5.282040000
H	0.674458000	6.483120000	4.098090000
C	2.567042000	5.057186000	2.747438000
H	2.355251000	4.088752000	3.213343000
H	1.711478000	5.323812000	2.121076000
H	3.451610000	4.956287000	2.111586000
C	3.985359000	5.674275000	4.703265000
H	3.744090000	4.733515000	5.206796000
H	4.885341000	5.520393000	4.099032000
H	4.205337000	6.426714000	5.466114000
H	1.691426000	7.133714000	5.387329000
H	2.673615000	11.406115000	2.759702000
H	0.449429000	9.073797000	3.634586000
H	-1.923892000	8.878717000	2.910388000

Int-1

C	4.526313000	10.373752000	5.367980000
C	5.820986000	10.535626000	4.852009000
C	6.787195000	9.506533000	4.971601000
H	6.502252000	8.566170000	5.433329000
C	8.083235000	9.692747000	4.509349000
H	8.802218000	8.884054000	4.613198000
C	8.468995000	10.896803000	3.916349000
H	9.484222000	11.035657000	3.557695000
C	7.523041000	11.915559000	3.783900000
H	7.801394000	12.856693000	3.316591000
C	6.221793000	11.746744000	4.237693000
H	5.496948000	12.546904000	4.123405000
C	3.360480000	10.198312000	5.770254000
C	5.563756000	11.675150000	9.058760000
N	3.661832000	10.021189000	8.937886000
N	3.185283000	12.012233000	8.301709000
C	4.186092000	11.242022000	8.745942000
Si	2.025711000	10.643628000	8.512539000
Cl	1.058501000	11.050066000	10.329813000
C	6.587423000	11.521007000	8.123334000
H	6.371104000	11.089124000	7.152727000
C	7.874587000	11.946708000	8.437859000
H	8.665721000	11.830663000	7.703439000
C	8.139909000	12.519796000	9.680220000
H	9.145215000	12.851439000	9.922316000
C	7.114827000	12.669093000	10.613872000
H	7.318410000	13.112248000	11.583851000
C	5.824010000	12.249484000	10.306414000
H	5.024383000	12.355671000	11.033732000
C	3.107415000	13.434636000	7.936087000
C	1.791526000	13.587788000	7.173610000
H	1.790999000	12.972926000	6.269019000
H	0.936627000	13.295386000	7.794670000
H	1.651061000	14.633229000	6.886588000
C	3.084101000	14.297464000	9.199481000
H	4.026091000	14.215047000	9.748218000

H	2.942006000	15.347950000	8.927061000
H	2.264573000	13.996300000	9.858867000
C	4.271861000	13.824713000	7.027501000
H	5.223958000	13.831539000	7.562113000
H	4.347584000	13.128741000	6.187855000
H	4.097707000	14.831445000	6.636089000
C	4.241595000	8.752708000	9.404682000
C	5.304091000	8.270052000	8.417289000
H	5.651558000	7.274678000	8.710805000
H	4.886510000	8.216556000	7.408413000
H	6.167824000	8.938948000	8.401086000
C	4.824275000	8.910592000	10.809646000
H	5.153714000	7.934938000	11.179895000
H	5.686799000	9.581300000	10.813836000
H	4.068597000	9.303727000	11.497015000
C	3.079715000	7.759654000	9.434092000
H	3.437902000	6.781276000	9.764828000
H	2.300257000	8.085321000	10.132004000
H	2.638112000	7.643811000	8.439175000
C	1.167274000	9.974360000	7.066573000
C	-0.242710000	9.569080000	6.916758000
C	-1.062560000	9.301460000	8.020894000
H	-0.662734000	9.395134000	9.025887000
C	-2.387189000	8.905544000	7.847788000
H	-3.003120000	8.699143000	8.718880000
C	-2.919342000	8.770432000	6.567607000
H	-3.952643000	8.463814000	6.432410000
C	-2.110464000	9.032917000	5.459661000
H	-2.514801000	8.932232000	4.455881000
C	-0.789702000	9.427352000	5.632386000
H	-0.150537000	9.631440000	4.777521000
C	2.082312000	9.952211000	6.074326000

P-1 (compound 1)

C	4.673279000	9.880172000	4.327907000
C	5.639769000	10.971544000	4.440020000
C	6.987843000	10.853923000	4.042221000
H	7.341607000	9.911858000	3.634548000
C	7.879610000	11.910986000	4.190002000
H	8.913170000	11.779714000	3.878163000
C	7.469681000	13.124815000	4.746968000
H	8.171502000	13.945131000	4.866688000
C	6.138842000	13.260010000	5.145055000
H	5.794157000	14.195925000	5.579579000
C	5.243107000	12.207318000	4.993846000
H	4.206263000	12.316136000	5.302573000
C	3.480008000	9.893738000	5.077582000
C	5.317025000	11.118755000	9.313596000
N	3.682673000	9.324278000	8.622215000
N	2.877117000	11.305095000	8.760448000
C	3.992799000	10.598078000	8.916997000
Si	1.972078000	9.788859000	8.239674000
Cl	0.761090000	9.366464000	9.881170000
C	6.256608000	11.424139000	8.326109000
H	6.018856000	11.270218000	7.278058000
C	7.487256000	11.959764000	8.692551000
H	8.207473000	12.207705000	7.918901000
C	7.784102000	12.179603000	10.036857000
H	8.746366000	12.596172000	10.319557000
C	6.846097000	11.866792000	11.020185000
H	7.075559000	12.034597000	12.068063000
C	5.608018000	11.340658000	10.661610000
H	4.873841000	11.094758000	11.423275000
C	2.551505000	12.726226000	8.923620000
C	1.264345000	12.947534000	8.126006000
H	1.417837000	12.724863000	7.065990000
H	0.454126000	12.307733000	8.493284000
H	0.942995000	13.988057000	8.226090000
C	2.304979000	13.017325000	10.406452000
H	3.220691000	12.875615000	10.987948000
H	1.977368000	14.053893000	10.535196000
H	1.530417000	12.355262000	10.804561000
C	3.651173000	13.633935000	8.373587000
H	4.562785000	13.588978000	8.973097000
H	3.899068000	13.362985000	7.344007000
H	3.292646000	14.667824000	8.382511000
C	4.490697000	8.097229000	8.638702000
C	5.620846000	8.183184000	7.610178000
H	6.082498000	7.197775000	7.489402000
H	5.241683000	8.519822000	6.640316000
H	6.400067000	8.877337000	7.933247000
C	5.048241000	7.844960000	10.040744000
H	5.570019000	6.882806000	10.058903000
H	5.761850000	8.619196000	10.333982000
H	4.238840000	7.813819000	10.777083000
C	3.526014000	6.974580000	8.262002000
H	4.062440000	6.022811000	8.214488000
H	2.726054000	6.880456000	9.004288000
H	3.072948000	7.168083000	7.286352000
C	1.340372000	9.652097000	6.607434000
C	-0.043153000	9.810156000	6.164567000
C	-1.139612000	9.263723000	6.862514000
H	-0.959279000	8.691866000	7.767770000
C	-2.441543000	9.418834000	6.398895000
H	-3.261245000	8.978404000	6.961759000
C	-2.704396000	10.112455000	5.214910000
H	-3.722432000	10.225001000	4.852966000
C	-1.631092000	10.658091000	4.509058000
H	-1.808877000	11.207874000	3.587147000
C	-0.328782000	10.513655000	4.975969000
H	0.501708000	10.950702000	4.427851000
C	2.428177000	9.822163000	5.724324000
C	1.176075000	7.618699000	1.749699000
N	3.262546000	7.422421000	3.143125000
N	3.267819000	9.016229000	1.702369000
C	2.537554000	8.002028000	2.173440000
Si	4.521792000	8.714589000	3.017064000
Cl	6.184203000	8.015194000	1.981945000
C	0.090130000	8.005937000	2.537690000
C	-1.202183000	7.678419000	2.137803000
C	-1.407971000	6.952469000	0.966173000
H	-2.416418000	6.691635000	0.658827000
C	-0.320055000	6.559686000	0.185811000
H	-0.478723000	5.993555000	-0.726977000
C	0.973763000	6.897980000	0.570061000
H	1.824087000	6.605698000	-0.038942000
C	2.881036000	10.237560000	0.975515000
C	4.188039000	10.988686000	0.717063000
H	4.684138000	11.251126000	1.658574000
H	4.873337000	10.383979000	0.115285000
H	3.980606000	11.917485000	0.178555000
C	2.211694000	9.902908000	-0.356226000
H	1.233176000	9.438702000	-0.216817000
H	2.067908000	10.826410000	-0.925511000
H	2.840345000	9.229220000	-0.946967000

C	1.959780000	11.087554000	1.853256000
H	1.004738000	10.581802000	2.022534000
H	1.755878000	12.045131000	1.363392000
C	3.071718000	6.165715000	3.883454000
C	1.855227000	6.267077000	4.806907000
H	1.843382000	5.411692000	5.490069000
H	0.921894000	6.253344000	4.239567000
C	2.926384000	4.992082000	2.913868000
H	2.879750000	4.055900000	3.479076000
H	2.011307000	5.071431000	2.321441000
H	3.784096000	4.943425000	2.235345000
C	4.341411000	5.990647000	4.714044000
H	4.257561000	5.097339000	5.339044000
H	5.220004000	5.877800000	4.070333000
H	4.494926000	6.853950000	5.366635000
H	1.886984000	7.189653000	5.394261000
H	2.429237000	11.279836000	2.823136000
H	0.249141000	8.567775000	3.453269000
H	-2.040385000	7.993926000	2.751886000

P-2

Cl	5.470073000	7.210927000	2.150972000
C	0.888843000	6.576031000	2.327200000
N	2.783730000	8.158426000	2.679901000
N	2.803625000	6.442200000	3.978136000
C	2.102446000	7.088684000	3.006784000
Si	4.245313000	7.550912000	3.829506000
Cl	5.628801000	6.492776000	5.174557000
C	-0.383609000	7.091380000	2.569915000
H	-0.518405000	7.896332000	3.284486000
C	-1.484465000	6.564974000	1.899980000
H	-2.474209000	6.965260000	2.098144000
C	-1.316378000	5.532940000	0.978782000
H	-2.176576000	5.125739000	0.456061000
C	-0.042000000	5.026181000	0.726515000
H	0.095476000	4.225606000	0.006142000
C	1.059283000	5.541878000	1.401979000
H	2.054125000	5.146647000	1.216448000
C	2.414085000	5.306823000	4.857802000
C	2.583610000	5.750931000	6.316434000
H	1.909703000	6.585090000	6.533484000
H	3.604467000	6.056910000	6.540374000
H	2.321825000	4.921114000	6.980500000
C	3.290788000	4.098055000	4.522001000
H	3.124654000	3.792165000	3.483706000
H	3.027505000	3.258170000	5.173103000
H	4.350020000	4.320094000	4.655228000
C	0.949396000	4.892711000	4.700537000
H	0.724813000	4.457634000	3.726147000
H	0.266945000	5.723881000	4.886722000
H	0.749944000	4.130636000	5.459491000
C	2.558029000	9.156044000	1.619339000
C	1.221446000	9.874880000	1.831676000
H	1.172183000	10.746078000	1.170910000
H	1.134140000	10.224008000	2.865613000
H	0.369747000	9.234177000	1.598771000
C	2.607042000	8.507849000	0.232999000
H	2.511605000	9.282218000	-0.534969000
H	1.791347000	7.794018000	0.094401000
H	3.558503000	7.989606000	0.086978000
C	3.670980000	10.199229000	1.735938000
H	3.558503000	10.932187000	0.931977000
H	4.662679000	9.749805000	1.649921000
H	3.610104000	10.728024000	2.689665000
C	4.419467000	9.172617000	4.774176000
C	5.589677000	10.064470000	4.698504000
C	6.900429000	9.601317000	4.506661000
H	7.093714000	8.535992000	4.457425000
C	7.958336000	10.501295000	4.412359000
H	8.967785000	10.122390000	4.279635000
C	7.732025000	11.875589000	4.479933000
H	8.560711000	12.572333000	4.394170000
C	6.430783000	12.347863000	4.648222000
H	6.237526000	13.416267000	4.686511000
C	5.375304000	11.451541000	4.761502000
H	4.358642000	11.811389000	4.885531000
C	3.371691000	9.478176000	5.536109000
Cl	1.248728000	11.334478000	10.441812000
C	5.676685000	11.629579000	9.005935000
N	3.533504000	10.375831000	8.999362000
N	3.383397000	12.349342000	8.152945000
C	4.235388000	11.440732000	8.711616000
Si	1.917194000	11.291110000	8.429486000
Cl	0.304866000	12.608106000	7.760413000
C	6.654627000	11.273053000	8.077945000
H	6.367723000	10.860293000	7.116974000
C	7.999468000	11.454335000	8.387853000
H	8.754224000	11.177517000	7.658226000
C	8.370237000	11.985800000	9.621461000
H	9.420366000	12.126621000	9.859830000
C	7.391457000	12.332976000	10.552362000
H	7.674624000	12.743333000	11.516942000
C	6.045758000	12.153935000	10.247605000
H	5.279246000	12.425430000	10.968174000
C	3.589363000	13.787940000	7.836957000
C	2.996620000	14.078710000	6.454262000
H	3.559774000	13.547385000	5.682678000
H	1.949327000	13.791177000	6.383238000
H	3.072657000	15.151262000	6.249800000
C	2.903073000	14.627263000	8.919495000
H	3.340583000	14.409469000	9.899308000
H	3.043418000	15.692780000	8.709865000
H	1.831455000	14.423854000	8.964464000
C	5.064029000	14.191788000	7.772176000
H	5.557641000	14.173431000	8.744315000
H	5.630846000	13.565703000	7.080044000
H	5.099219000	15.220882000	7.401894000
C	3.932960000	9.079992000	9.578643000
C	5.017992000	8.406083000	8.734637000
H	5.174040000	7.385208000	9.097784000
H	4.718011000	8.350544000	7.684802000
H	5.972187000	8.932211000	8.798460000
C	4.407883000	9.267805000	11.023120000
H	4.592603000	8.287988000	11.475207000
H	5.336859000	9.839733000	11.070355000
H	3.642958000	9.783458000	11.611135000
C	2.696363000	8.177722000	9.585626000
H	2.949427000	7.234954000	10.078935000
H	1.868783000	8.635584000	10.133483000
H	2.363581000	7.949565000	8.571666000
C	1.430541000	9.926078000	7.197726000
C	0.155865000	9.196113000	7.198295000
C	-0.741714000	9.284165000	8.272612000
H	-0.506705000	9.913118000	9.123814000
C	-1.937055000	8.572473000	8.264147000

H	-2.614239000	8.656106000	9.109118000
C	-2.267007000	7.762345000	7.180430000
H	-3.202148000	7.210237000	7.171888000
C	-1.387707000	7.671329000	6.100199000
H	-1.643642000	7.046286000	5.248812000
C	-0.193691000	8.379233000	6.109098000
H	0.493652000	8.317564000	5.269156000
C	2.382729000	9.681520000	6.299586000

LSi-Dimer

C	6.944986000	12.096930000	6.049014000
N	9.216574000	10.990528000	6.068372000
N	9.039920000	12.691185000	7.341206000
C	8.363819000	11.943338000	6.454173000
Si	10.524003000	11.509633000	7.340083000
C	5.941502000	11.403008000	6.732215000
H	6.211391000	10.740279000	7.549274000
C	4.608790000	11.547141000	6.357486000
H	3.836192000	11.003926000	6.893778000
C	4.268236000	12.381661000	5.293531000
H	3.228429000	12.492310000	5.000109000
C	5.265236000	13.071488000	4.605921000
H	5.005614000	13.721568000	3.775540000
C	6.598464000	12.931587000	4.982976000
H	7.375169000	13.475727000	4.454107000
C	8.558376000	13.641818000	8.343842000
C	7.790333000	12.914083000	9.452386000
H	6.858878000	12.488145000	9.068744000
H	8.403071000	12.105101000	9.862486000
H	7.537076000	13.607801000	10.261738000
C	9.817438000	14.280511000	8.931649000
H	10.407257000	14.746788000	8.137328000

Optimised coordinates of compound $\mathbf{2}^{\bullet-}$
q = -1, S = ½
E_{hf} = -4423.4246107

17	5.403719000	7.282406000	2.062826000
6	0.991908000	6.442951000	2.461821000
7	2.771636000	8.136327000	2.768307000
7	2.954531000	6.420677000	4.043449000
6	2.181728000	7.036262000	3.114245000
14	4.343868000	7.593243000	3.869201000
17	5.854789000	6.463875000	5.000997000
6	-0.302782000	6.875442000	2.708929000
1	-0.481645000	7.655932000	3.435871000
6	-1.367269000	6.287057000	2.043721000
1	-2.375900000	6.622695000	2.252548000
6	-1.143508000	5.275462000	1.121965000
1	-1.977725000	4.819303000	0.602423000
6	0.152923000	4.848942000	0.866623000
1	0.335774000	4.062092000	0.144705000
6	1.217274000	5.427148000	1.536742000
1	2.233162000	5.102073000	1.346175000
6	2.634040000	5.284288000	4.932451000
6	2.917998000	5.714884000	6.371539000
1	2.273409000	6.552969000	6.637851000
1	3.952296000	6.015585000	6.509459000
1	2.702449000	4.886429000	7.050024000
6	3.473916000	4.073636000	4.531421000
1	3.216391000	3.758769000	3.517260000
1	3.272768000	3.240494000	5.209254000
1	4.536203000	4.305381000	4.565226000
6	1.162194000	4.880372000	4.895606000
1	0.857065000	4.441734000	3.947991000
1	0.508654000	5.719491000	5.130730000
1	1.016411000	4.127592000	5.672250000
6	2.405994000	9.163506000	1.792190000
6	1.131163000	9.878623000	2.240498000
1	0.979372000	10.770517000	1.628542000
1	1.219591000	10.184946000	3.284059000
1	0.251403000	9.246884000	2.130464000
6	2.237077000	8.577530000	0.391591000
1	2.072593000	9.387661000	-0.322487000
1	1.385971000	7.899528000	0.332639000
1	3.138915000	8.036982000	0.099979000
6	3.530298000	10.195928000	1.766600000
1	3.286119000	10.965976000	1.032279000
1	4.482542000	9.744251000	1.493264000
1	3.644909000	10.667674000	2.740849000
6	4.475188000	9.190217000	4.771106000
6	5.589879000	10.103662000	4.654519000
6	6.894242000	9.701754000	4.306066000
1	7.102651000	8.651543000	4.153470000
6	7.918677000	10.622579000	4.184937000
1	8.912888000	10.271705000	3.928496000
6	7.694239000	11.981652000	4.382031000
1	8.500133000	12.698049000	4.276649000
6	6.408527000	12.400335000	4.705689000
1	6.202236000	13.456342000	4.844466000
6	5.383897000	11.484774000	4.847793000
1	4.383178000	11.812491000	5.101915000
6	3.351689000	9.544226000	5.469515000
17	1.175503000	11.294904000	10.336325000
6	5.627118000	11.537057000	8.998497000
7	3.451721000	10.372554000	8.898270000
7	3.383948000	12.385246000	8.162781000
6	4.185978000	11.417797000	8.676101000
14	1.862515000	11.369609000	8.323295000
17	0.360413000	12.893863000	7.834104000
6	6.603274000	11.178655000	8.078523000
1	6.318959000	10.820409000	7.097980000
6	7.942736000	11.297292000	8.414055000
1	8.693442000	11.023992000	7.683027000
6	8.311641000	11.763759000	9.667102000
1	9.359996000	11.855801000	9.925810000
6	7.335390000	12.108787000	10.591935000
1	7.616830000	12.468257000	11.574600000
6	5.995650000	11.995184000	10.259125000
1	5.226761000	12.265253000	10.973377000
6	3.673472000	13.807323000	7.885773000
6	3.103892000	14.169695000	6.513745000
1	3.645077000	13.637746000	5.731170000
1	2.048891000	13.927103000	6.433990000
1	3.228945000	15.241782000	6.344879000
6	3.042997000	14.662691000	8.985531000
1	3.480439000	14.406224000	9.953267000
1	3.236003000	15.720836000	8.791573000
1	1.967519000	14.506388000	9.039223000
6	5.163335000	14.135973000	7.829642000
1	5.653334000	14.070374000	8.798530000
1	5.695900000	13.500325000	7.124854000
1	5.250005000	15.168024000	7.483588000
6	3.796472000	9.042563000	9.410694000
6	4.879674000	8.377247000	8.564665000
1	4.972156000	7.329186000	8.857599000

1	4.622283000	8.414995000	7.506258000
1	5.851964000	8.846978000	8.703593000
6	4.237876000	9.133036000	10.871678000
1	4.370430000	8.125542000	11.272803000
1	5.185097000	9.661865000	10.972861000
1	3.478433000	9.646387000	11.463450000
6	2.535170000	8.186303000	9.340326000
1	2.756197000	7.196344000	9.743603000
1	1.724694000	8.626358000	9.920741000
1	2.197658000	8.077100000	8.312758000
6	1.349696000	10.142496000	7.030567000
6	0.077668000	9.464163000	6.989517000
6	-0.971159000	9.750596000	7.885957000
1	-0.847057000	10.538255000	8.615877000
6	-2.161751000	9.051293000	7.852752000
1	-2.942487000	9.308549000	8.560704000
6	-2.367569000	8.026440000	6.935411000
1	-3.299312000	7.473772000	6.918038000
6	-1.344684000	7.722644000	6.044341000
1	-1.482223000	6.916131000	5.331220000
6	-0.154308000	8.424689000	6.063857000
1	0.644835000	8.178946000	5.372339000
6	2.363162000	9.814381000	6.170155000

H	9.553502000	15.035145000	9.678948000
H	10.440456000	13.520882000	9.416977000
C	7.680567000	14.732885000	7.725946000
H	8.193740000	15.220812000	6.892096000
H	6.731223000	14.335088000	7.361055000
H	7.458914000	15.492279000	8.482990000
C	9.149032000	10.028577000	4.968326000
C	7.845458000	9.225266000	4.979925000
H	7.907894000	8.423254000	4.237026000
H	7.682910000	8.772229000	5.963102000
H	6.979291000	9.843645000	4.734525000
C	9.309234000	10.762122000	3.632906000
H	9.308862000	10.047351000	2.802913000
H	8.488428000	11.468247000	3.475224000
H	10.256530000	11.310716000	3.618038000
C	10.326121000	9.072579000	5.163905000
H	10.354344000	8.341559000	4.350227000
H	11.274364000	9.621783000	5.159971000
H	10.236536000	8.535934000	6.113691000
C	13.588898000	15.779596000	7.238666000
N	13.342015000	13.268220000	7.110553000
N	11.948076000	14.364873000	5.927950000
C	12.965100000	14.509548000	6.792982000
Si	12.178455000	12.483926000	5.835073000
C	14.691200000	16.297961000	6.551991000
H	15.094758000	15.755349000	5.702084000
C	15.277014000	17.491402000	6.964087000
H	16.132915000	17.886331000	6.424596000
C	14.769657000	18.173969000	8.069111000
H	15.228230000	19.104132000	8.391690000
C	13.674363000	17.658786000	8.760255000
H	13.276303000	18.185663000	9.622517000
C	13.084536000	16.467287000	8.345910000
H	12.225327000	16.069650000	8.877632000
C	11.365807000	15.317174000	4.981674000
C	12.360313000	15.644392000	3.862497000
H	13.219133000	16.199011000	4.251163000
H	12.720454000	14.720392000	3.399229000

H	11.880995000	16.259295000	3.092690000
C	10.149225000	14.604475000	4.389885000
H	9.461672000	14.312132000	5.188467000
H	9.627743000	15.255904000	3.681871000
H	10.458485000	13.697621000	3.858145000
C	10.909493000	16.605315000	5.671125000
H	10.245736000	16.383548000	6.511954000
H	11.753420000	17.190268000	6.042981000
H	10.359660000	17.224874000	4.955078000
C	14.223614000	12.773191000	8.167840000
C	15.592032000	13.460050000	8.150702000
H	16.259697000	12.954553000	8.856201000
H	16.037997000	13.401917000	7.152661000
H	15.528242000	14.510529000	8.442702000
C	13.549950000	12.964753000	9.530431000
H	14.177813000	12.552382000	10.327622000
H	13.387447000	14.026519000	9.739004000
H	12.584636000	12.448595000	9.546870000
C	14.411510000	11.279290000	7.902379000
H	15.039949000	10.836198000	8.680826000
H	13.445718000	10.761632000	7.909946000
H	14.889007000	11.114987000	6.931228000

5. References

[1] (a) J. P. Perdew, K. Burke, M. Ernzerhof, *Phys. Rev. Lett.* **1996**, *77*, 3865; (b) J. P. Perdew, K. Burke, M. Ernzerhof, *Phys. Rev. Lett*. **1997**, *78*, 1396; (c) C. Adamo, V. Barone, *J. Chem. Phys.* **1999**, *110*, 6158.

[2] *Gaussian 16*, *Revision A.03*, M. J. Frisch, et al. Gaussian, Inc., Wallingford CT. **2016**.

[3] a) F. Weinhold, C. Landis, *Valency and Bonding, A Natural Bond Orbital Donor – Acceptor Perspective,* Cambridge University Press, Cambridge, **2005**; b) C. R. Landis and F. Weinhold, *"The NBO View of Chemical Bonding", in, G. Frenking and S. Shaik (eds.), The Chemical Bond: Fundamental Aspects of Chemical Bonding.* Wiley, **2014**, pp. 91-120.

[4] K. B. Wiberg, *Tetrahedron* **1968**, *24*, 1083.

[5] (a) E. D. Glendening, C. R. Landis, F. Weinhold, *J. Comput. Chem*. **2013**, *34*, 1429. (b) Atoms in Molecules", R.F.W. Bader, *Acounts of Chemical Research*, **1985**, *18*, 9-15. (c) AIMAll (Version 19.10.12), Todd A. Keith, TK Gristmill Software, Overland Park KS, USA, 2019 (aim.tkgristmill.com).

[6] (a) R. F. W. Bader, "Atoms in Molecules: A Quantum Theory," Oxford University Press, Oxford 1990. (b) T. A. Keith, AIMAll (Version 15.05.18), TK Gristmill Software, Overland Park, KS 2015.

[7] T. Ziegler, A. Rauk, *Theor. Chim. Acta,* **1977**, *46*, 1.

[8] (a) M. Mitoraj, A. Michalak, *Organometallics* **2007**, *26*, 6576; (b) M. Mitoraj, A. Michalak, *J. Mol. Model.* **2008**, *14*, 681.

[9] (a) *ADF2017*, SCM, Theoretical Chemistry, Vrije Universiteit, Amsterdam, The Netherlands, http://www.scm.com; (b) G. te Velde, F.M. Bickelhaupt, E. J. Baerends, C. F. Guerra, S. J. A. van Gisbergen, J. G. Snijders, T. Ziegler, *J. Comput. Chem.* **2001**, *22*, 931.

[10] (a) E. van Lenthe, E. J. Baerends, *J. Comput. Chem*. **2003**, *24*, 1142; (b) E. van Lenthe, E. J. Baerends, J. G. Snijders, *J. Chem. Phys*. **1993**, *99*, 4597; (c) E. van Lenthe, E. J. Baerends, J. G. Snijders, *J. Chem. Phys*. **1994**, *101*, 9783.

[11] (a) G. Frenking, F. M. Bickelhaupt, *The Chemical Bond 1. Fundamental Aspects of Chemical Bonding*, chap. The EDA Perspective of Chemical Bonding, 121. Wiley-VCH: Weinheim, **2014**; (b) L. Zhao, M. von Hopffgarten, D. M. Andrada, G. Frenking, *WIREs Comput. Mol. Sci.* **2018**, *8*, 1345; (c) L. Zhao, M. Hermann, W. H. E. Schwarz, G. Frenking, *Nat. Rev. Chem.* **2019**, *3*, 48; (d) W. Yang, K. E. Krantz, L. A. Freeman, D. Dickie, A. Molino, G. Frenking, S. Pan, D. J. D. Wilson, R. J. Gilliard Jr., *Angew. Chem. Int. Ed.*, **2020**, *59*, 3850; (e) G. Deng, S. Pan, G. Wang, L. Zhao, M. Zhou, G. Frenking, *Angew. Chem. Int. Ed*., **2020**, *59*, 10603; (f) S. Pan, G. Frenking, *Angew. Chem. Int. Ed*., **2020**, *59*, 8756; (g) L. Zhao, S. Pan, M. Zhou, G. Frenking, *Science*, **2019**, *365*, eaay5021; (h) R. Saha, S. Pan, P. K. Chattaraj, G. Merino, *Dalton Trans*. **2020**, *49*, 1056; (i) S. M. N. V. T. Gorantla, P. Parameswaran, K. C. Mondal, *J. Comput. Chem.* **2021**, *42* (16), 1159.

(12) J. Andrés, P. W. Ayers, R. A. Boto, R. Carbó-Dorca, H. Chermette, J. Cioslowski, J. Contreras-García, D. L. Cooper, G. Frenking, C. Gatti, F. Heidar-Zadeh, L. Joubert, A. Martín Pendás, E. Matito, I. Mayer, A. J. Misquitta, Y. Mo, J. Pilmé, P. L. A. Popelier, M. Rahm, E. Ramos-Cordoba, P. Salvador, W. H. E. Schwarz,

S. Shahbazian, B. Silvi, M. Solà, K. Szalewicz, V. Tognetti, F. Weinhold, E. L. Zins, *J. Comput. Chem*. **2019**, *40*, 2248.

[13] K. B. Beć, J. Grabska, Y. Ozaki, J. P. Hawranek, C. W. Huck, *J. Phys. Chem. A*, **2017**, *121*, 1412–1424.

[14] R. V. Singh, P. Chaudhary, S. Chauhan, M. Swami, *Spectrochim Acta A Mol. Biomol. Spectrosc.* **2008**, *72*, 260–268.

[15] X. Kong, Z. Wu, M. Strømme, C. Xu, *J. Am. Chem. Soc.* **2023**, *146*, 742–751.

[16] K. I. Hadjiivanov, D. A. Panayotov, M. Y. Mihaylov, E. Z. Ivanova, K. K. Chakarova, S. M. Andonova, N. L. Drenchev, *Chem. Rev.* **2020**, *121*, 1286–1424.

[17] J. S. Böke, J. Popp, C. Krafft, *Sci. Rep.*, **2022**, 12, 18785.

[18] R. D. Kross, V. A. Fassel, M. Margoshes, *J. Am. Chem. Soc.* **1956**, *78*, 1332–1335.

[19] A. L. Smith, *J. Chem. Phys.* **1953**, *21*, 1997–2004.

[20] Bruker, *SAINT*, *V8.40* and *V8.41*, Bruker AXS Inc., Madison, Wisconsin, USA.

[21] L. Krause, R. Herbst-Irmer, G. M. Sheldrick, D. Stalke, *J. Appl. Cryst.* **2015**, *48*, 3–10, doi:10.1107/S1600576714022985.

[22] G. M. Sheldrick, *Acta Cryst.* **2015**, *A71*, 3–8, doi:10.1107/S2053273314026370.

[23] G. M. Sheldrick, *Acta Cryst.* **2015**, *C71*, 3–8, doi:10.1107/S2053229614024218.

[24] C. R. Groom, I. J. Bruno, M. P. Lightfoot, S. C. Ward, *Acta Cryst.* **2016**, *B72*, 171–179, doi:10.1107/S2052520616003954.

[25] D. Kratzert, *FinalCif*, *(Bruker Edition)*, https://dkratzert.de/finalcif.html.
